# Tuning Size and Properties of Zinc Ascorbate Metal-Organic Framework via Acid Modulation

**DOI:** 10.3390/molecules28010253

**Published:** 2022-12-28

**Authors:** Tia Kristian Tajnšek, Nataša Zabukovec Logar, Matjaž Mazaj

**Affiliations:** 1 National Institute of Chemistry, Hajdrihova 19, 1000 Ljubljana, Slovenia; 2 Faculty of Inorganic Chemistry and Technology, University of Ljubljana, Večna pot 113, 1000 Ljubljana, Slovenia; 3 School of Science, University of Nova Gorica, Vipavska 13, 5000 Nova Gorica, Slovenia

**Keywords:** metal-organic framework, acid modulators, coordination modulation, defects

## Abstract

One of the biggest advantages of MOFs is the possibility of modifying their properties and tuning their inherent activity (i.e., sorption, storage, catalytic activity etc.). Textural properties can be tuned by manipulating process and compositional parameters, among which, the effect of additives can be even further distinguished among them based on the way they affect these properties. Beyond the effect that additives have on the size and morphology of nanoMOFs, there is also an effect on properties via creating point defects—missing linker and missing node defects. In this study, we investigated the effect of four monotopic acid modulators—formic, acetic, dichloroacetic and propionic acid, their concentration and the heating type (conventional and microwave—MW) on the size, morphology and textural properties of a recently discovered bioNICS1. It was confirmed that the proposed seesaw model for the controlled size of nanoMOF crystals is less applicable in the case of MW-assisted synthesis, in comparison to conventional heating. In the case of formic acid- and propionic acid-modified materials, we demonstrated that the type of additive plays a different role in crystal growth and generation of defects, implying high tunability being crucial for a material’s structure–property performance optimization.

## 1. Introduction

Metal-organic frameworks (MOFs) are crystalline and generally porous materials consisting of metal ions coordinated to organic molecules. Large pore volume and high surface-to-volume ratio are properties commonly attributed to these materials. MOFs are already well established in the various fields of science, such as catalysis, gas storage and separation, sensing or electrochemistry and they are increasingly gaining interest in biomedicine applications as well [1,2,3,4,5].

One of the biggest advantages of MOFs is the possibility of modification of their properties and the tuning of their inherent activity (i.e., sorption [6], catalytic [7], electro-magnetic [8], optic activity [9] etc.). For this, we usually have to manipulate the size and morphology of the crystals, porosity of the structure, functionalize linkers or create defects in the framework. We do so by controlling compositional (molar ratio, metal source, linker concentration, addition of additives/agents/templates) and process parameters, or we post-synthetically treat the material [10].

For the purpose of tuning size and morphology of MOF crystals, most commonly used additive species can be further distinguished among themselves based on the way they affect these properties [10]:

(i) *Blocking agents* slow down or *manipulate crystal growth in a certain direction* by adsorption on a certain crystal face and so control crystal morphology. Effects of pyridine on the shape of In-MIL-53 [11] and acetic acid on the [{Cu2(ndc)2-(dabco)}n] [12] serve as examples of such shape manipulation.
(ii) *Surface capping agents* usually *stop or inhibit further crystal growth*. Specific amounts of acetic acid [13] and p-perfluoromethybenzoic acid [14] were used as growth inhibitors in the synthesis of corresponding MOFs.
(iii) The *Modulators* role is to *control size and morphology* by competing with organic linkers in order to coordinate with the metal ions during nucleation and growth processes [10]. Most commonly used additives are mono-carboxylic acids and/or their salts. There are a wide range of such modulators present in the literature: acetic, formic and propionic acids [15]; lauric acid [16]; benzoic acid [17].

Considering the example of acetic acid being used by different researchers as a blocking agent, surface capping agent and modulator, the chosen term for a specific additive therefore mostly depends on the *way* that it affects the framework. We must further acknowledge the fact that this influence could change based upon additives’ concentrations.

**Figure 1:**
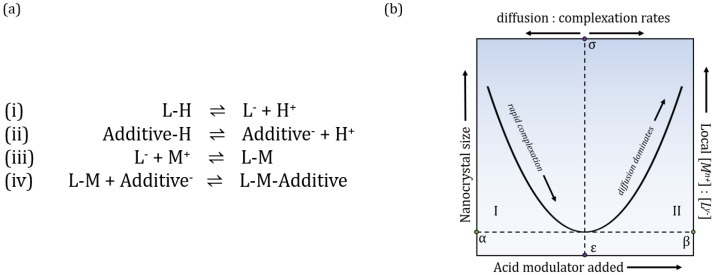
(**a**) Chemical equilibria controlling crystal growth and termination; deprotonation of linker (i) and additives (ii), complexation (iii) between metal ions and linkers = growth phase, (iv) competition between linker and additive for coordination sites on metal ions = termination phase. (**b**) Relationship between nanocrystal size, local metal-linker ratios, amount of modulator added and diffusion and complexation rates can be schematically represented in a seesaw model, with Regime I on the left and Regime II on the right side.

The MOF particle’s formation and growth was proposed to be kinetically controlled by chemical parameters that will trap its growth in nano size limits by an alternative of the LaMer model [18]. The “entrapment” is dependent on competition between four chemical equilibria (Figure 1a). According to the proposed model, this process is continuous until the local concentration of metal ions is depleted and so the particle growth stops. Presence of the most commonly used additives—carboxylic acids—can impede linker deprotonation, so complexation (iii) (relative to metal diffusion) is slowed down and larger nano-MOF sizes are reachable. However, to produce smaller sizes, these additives must also act as surface capping ligands and terminate particle growth, affecting equilibria (iv). Relationship between size, metal-linker ratios, amount of added modulator and diffusion: complexation rates can be presented in a seesaw relationship (Figure 1b), which suggests that synthesis conditions which would allow the formation of nanosized particles and identify conditions that would favor the synthesis of larger microscopic crystals.

Beyond the effect that additives have on the size and/or morphology of nanoMOF, there is also an evident effect on properties via creating point defects—missing linker or missing node [19]. Defects can be introduced into MOFs either (i) during synthesis with *modulator*, mixed linker or *fast crystal growth* approach, or (ii) post synthetically by mechanical, acid/base, solvent assisted ligand exchange and harsh activation treatment. In the case of modulator approach, mono-carboxylic acids are used for reducing the speed of crystallization to obtain higher degrees of crystallinity; however, larger quantities can facilitate the formation of defects. From this approach follows the notion that fast crystallization could similarly lead to formation of point defects. One of the most favorable synthetic pathways is microwave assisted synthesis [19]. Incorporation of defects in the original structures offers an additional pathway of tuning inherent (micro-porosity) or introducing new (active catalytic sites, charge transport) properties of parent structures.

Herein, we present an in situ modulation study of porous zinc ascorbate MOF material, recently developed in our lab (bioNICS-1), during the solvothermal process under conventional and microwave heating [20]. The effects of type and concentration of monotopic additives on crystallite size were systematically explored. Furthermore, the generation of defects and acid sites were studied on the selected modulated structures. BioNICS-1, composed of biocompatible components (zinc and ascorbic acid—ASC) with an open porous framework, represents a high potential in the medical field as a drug delivery system. The morphology tuning and controlled generation of acid sites is, therefore, of high relevance for biomedical use.

## 2. Results and Discussion

Acids were chosen based upon their already established frequent use as monotopic additives in the synthesis of MOFs, as well as their acidity and molecular size. These parameters have, according to the literature, the most significant effect on crystal growth dynamics, morphology and possible generation of defects [21,22]. With a bio-application in mind, less toxic candidates—presented in Table 1—were chosen from the set. For further clarification: lowercase abbreviation is used for samples obtained by conventional synthesis in an oven (i.e., aa, fa, pa, daa) and upper case is used for samples obtained by microwave synthesis (i.e., AA, FA, PA, DAA).

### 2.1. Textural Properties

**Figure 2:**
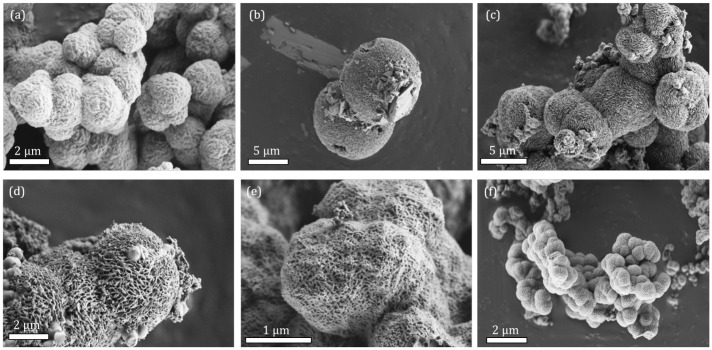
SEM micrographs of bioNICS1 with increasing amount of dichloroacetic acid as an additive. (**a**) 1.5 mol; (**b**) 2 mol; (**c**) 3 mol; (**d**) 4 mol; (**e**) 6 mol; (**f**) pristine bioNICS-1 with no additive.

Modified materials take the shape of quasi-spherical particles where crystal domains are visible, resulting in a rough surface. When lower concentrations of additives were used, the surface of particles was less defined but still distinguishable (Figures S1–S4). Crystal domains become clearly visible on the surface of the particles where higher concentrations of additives were used, and their sizes match the calculated ones. This is particularly true in the case of MW synthesis where surface and morphology stay more or less the same. In the conventional synthesis, however, there is clearly visible effect of acetic and dichloroacetic acid on the surface roughness of the particles. In the case of acetic acid (aa), the particle surface becomes smooth, and we cannot distinguish any crystalline domains (Figure S1). In the case of dichloroacetic acid (daa), when added in sufficient amounts, crystals grow in lamellar shape in a lateral direction (Figure 2). With even larger addition of daa, particle surface again changes shape into a thick spider web-like pattern with visible voids. Such an effect makes the daa a so-called blocking agent, restricting the growth of domains in a certain direction, though it seems its effect is confined to the surface of the particles.

PXRD diffraction patterns of pristine materials (Figure S5) confirm that bioNICS1 material crystalizes in both conventional and microwave (MW) heating conditions. PXRD analysis also confirms that all materials, regardless of the additive used (where the synthesis was successful), crystalize in the same crystal structure with different degrees of crystallinity (Figures S6–S9). Sherrer calculations were conducted on the first four characteristic peaks to determine the crystal sizes of all of the samples; however, only certain materials were used for further analysis. Selection was based upon the calculated size and the synthesis yield.

**Figure 3:**
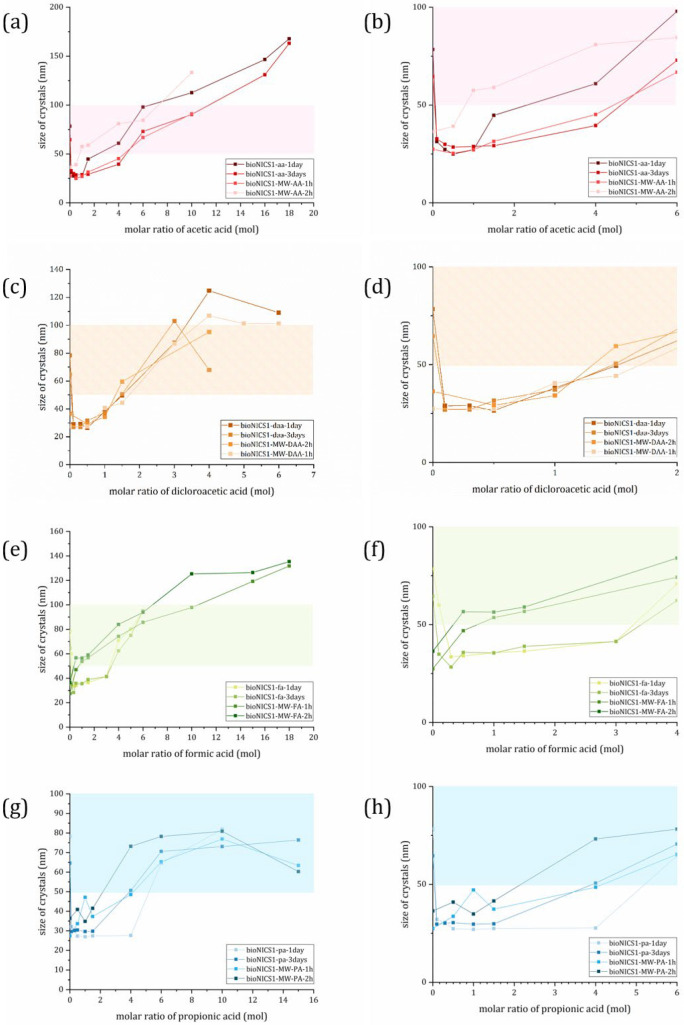
Calculated size of crystal domains plotted against molar addition of used additive; (**a**) and (**b**) acetic acid; (**c**) and (**d**) dichloroacetic acid; (**e**) and (**f**) formic acid; and (**g**) and (**h**) propionic acid. Colored ribbon represents a region of 50–100 nm sizes. Graphs on the right side of figure show lower molar additions of acids to emphasize seesaw trend. Error bars are omitted for clarity. Graphs with error bars are found in Supplementary Figure S10.

When the size of the crystals is plotted against the molar addition of the acid (Figure 3), their effect on the development of the size is clearly visible, and in the case of conventional heating confirms the entire range of the seesaw model proposed by Marshall et.al. [18]. In the case of MW synthesis, the minimal size is apparently reachable without the additive. With further addition, we increase the sizes and thus follow the Regime II of the proposed seesaw model (Figure 1b). We can also confirm that all four chosen acids can act as surface capping agents in lower concentrations.

The curvature of the slope in the Regime II is proportional to the additives’ acidity. The more acidic the additive is, the larger particles produced at fixed equivalents, following PA < AA < FA < DAA order. This was confirmed in all but two of our cases. MW synthesis with the addition of AA and FA does not follow this predicament. We suspect that this is due to the already present acetate ion from the metal precursor used (Zn-acetate); however, the specific mechanism of this effect is not the subject of this research. An extreme limit at the far right of the curve in the seesaw model involves particle growth over a longer period, due to sluggish metal–ligand complexation; this puts MOF crystal growth in a different regime that is determined by thermodynamics. Therefore, the “cut off” point was set up to the acid addition, at which the size no longer follows the quadratic curvature of the model (see Figure S11 for clarification of methodology used).

**Figure 4:**
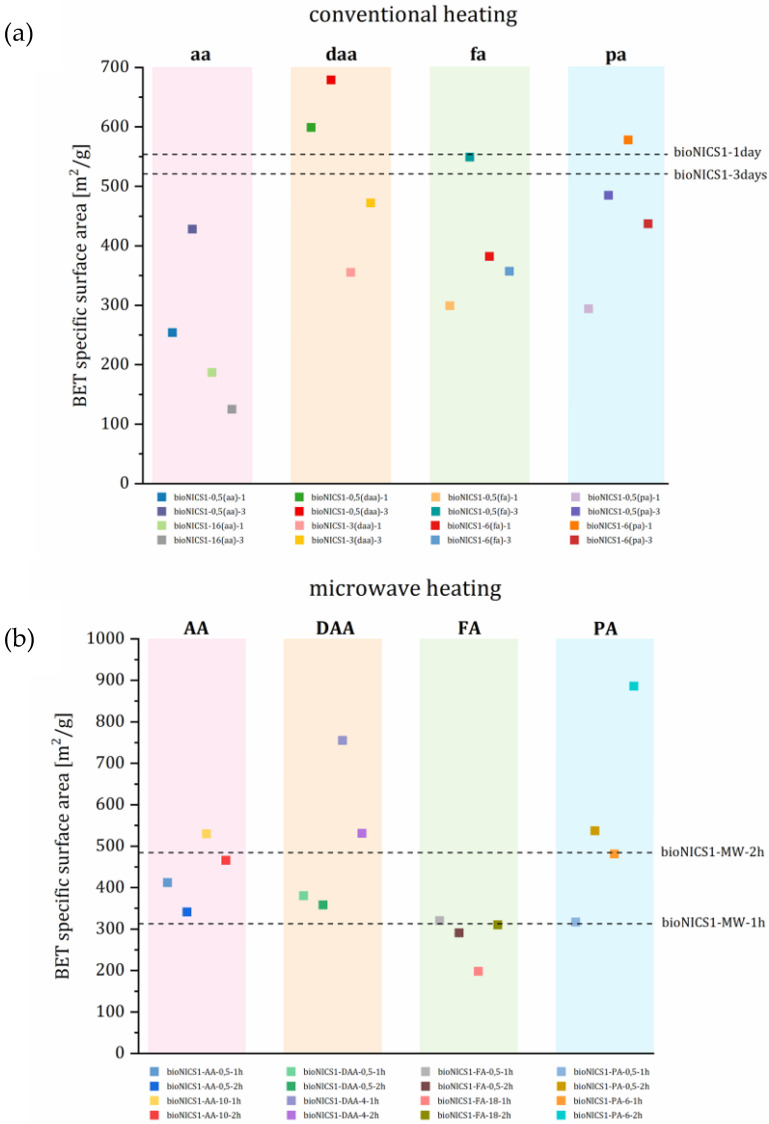
BET surface area of selected materials (**a**) conventional and (**b**) microwave heating arranged according to acid used. Colors of the squares are correspondent to the isotherms and pore size distribution presented in Supplementary. Dotted lines represent the BET value of pristine materials.

Change in specific surface area (BET) is generally governed by the degree of crystal structure order and plays a crucial role for the accessibility of sorption sites for guest molecules. The presence of an additive within the MOF framework is thus expected to have an impact on specific surface area as well. All adsorption isotherms show a type I shape typical for microporous materials (Figures S12a–S15a). Pristine bioNICS-1 formed under microwave heating have significantly lower BET (313 m^2^/g and 485 m^2^/g) in comparison to the products that crystallize under conventional heating (554 m^2^/g and 521 m^2^/g). Faster crystallization under microwave conditions apparently causes a higher degree of disorder of the crystal structure framework, resulting in lower accessibility of micropores for gaseous molecules. Under conventional solvothermal conditions, additives seem to have a deteriorating effect on the specific surface area, possessing generally lower BET values as pristine material (Figure 4). This implies that conventional heating may promote the monotopic acids to act as surface capping or blocking agents rather than modulators, since they clearly have an impact on morphology, agglomeration and size of the crystals. On the other hand, binding on the surface of the crystallites partially blocks the accessibility to micropores. The effect of agglomeration and/or surface roughness can be additionally proved by the occurrence of interparticle mesoporosity (Figure S12b–S15b).

In contrast to conventional heating, microwave heating, in most cases, yields products with higher specific surface areas with respect to the pristine material. Under microwave heating conditions, monotopic acids clearly undergo different a binding mechanism to the MOF framework, when compared to the conventional heating process. The effect of BET value increment can be a consequence of partial ligand replacement, causing the occurrence of either ligand or metal node deficiencies. This could cause the formation of additional vacancies within the framework structure contributing to the materials’ micropore volume and increase in specific surface area [19]. This implies that acid additives act also as modulators under microwave heating. The presence of acid additives has an impact on a bulk crystal structure rather than on an outer surface modification, which is indicated by a less pronounced occurrence of interparticle mesoporosity than in the case of conventional heating (Figures S12b–S15b).

### 2.2. In Search of Defects

To confirm assumptions of different binding mechanisms under conventional and microwave heating, a more in-depth study of possible structural defects was performed on the selected materials. As we described above, the use of additives in the corresponding coordination modulation technique can result in additives acting as surface capping agents. This means the additive will terminate the crystal growth by coordinating to the metal site where a polydentate linker would normally be found. The capping agent’s lack of binding sites prevents further assembly of the MOF. This will not only affect the crystal size but surface chemistry as well. The functionality of a capping agent is, therefore (theoretically), confined to the surface with the potential to alter the bulk properties of the MOF particles. This, however, is not always the case. Since MOFs are very individualistic materials, coordination modulation in some combinations of MOF-modulator-solvent result not only in selective surface capping, but in generation of defects throughout the framework as well [23].

#### 2.2.1. Surface Properties

For medical use, the arbitrary size range of nanoparticles is set to 1–100 nm in one dimension, but larger particles are also being studied. Shape, size and surface properties of nanoparticles will affect their biodistribution, cellular uptake, metabolism, clearance and so on. Multiple studies report good cellular uptake and intracellular delivery when particles ~50 nm in diameter are used. However, the kidneys might more readily clear these particles. The problem with larger particles (>150 nm) is opsonization and activation of the human complement system [24,25]. For Zeta potential (ζ-potential) measurements, a suitable size range was set between 50 and 100 nm (represented by a colored ribbon in Figure 3), since sizes outside this range are a little less desirable in the biomedical field.

It was previously determined that ζ-potential of MOF nanoparticles can be affected by varying the number of defects introduced in the samples, or more accurately on the samples (accomplished by increasing the concentration of a chosen modulator or using modulators with different acid strengths) [26]. ζ-potential of selected materials in our study spanned from roughly 0 to 40 (Figure S16), which was unexpected considering that surface confined capping agents should result in an increase in the ζ-potential to the (+) side. With these results, we would suggest that particles do not have surface defects per se, but rather dangling side chains of annexed linkers. This would explain the negative charge of Zeta potential.

All but one of the samples cross the threshold value of |20 mV| in the physiological pH range of 7.5–7.45, making them (theoretically) less prone towards aggregation. The pristine material reaches only −10 mV in this pH range [20], so we consider this a successful surface modification. It is apparent that regardless of the type of the modulator used and its concentration, Zeta potential gets lower. Size analysis and polydistribution index (PDI) are, however, not obtainable from DLS measurement due to sedimentation of the sample; whether or not agglomeration occurs, and if deflocculating is possible, was not addressed.

#### 2.2.2. Bulk Defects

When examining defects, one firstly looks at experiments conducted on the UiO-66 material since defects in that MOF have been the most widely and thoroughly studied [27]. Because added acids, that can act as modulators (modulator approach), and fast crystallization (MW synthesis) are both known as defect engineering procedures [19], some initial insights could be obtained in our case. MOFs are already established as prominent gas absorbers in the biomedical field as well. They present a suitable carrier for therapeutic gases such as NO [28]. As NO binds to the unsaturated metal sites (or Lewis acid sites), generation of such sites via point defects presents a great research interest in this field.

Following the principle of TG analysis presented in the UiO-66 study [27], where the magnitude of weight loss at the framework decomposition weight loss step is inversely proportional to modulator concentration and consequently the linker deficiency, we decided to follow similar approach. TG curves were normalized to present inorganic residue (ZnO) = final weight at 100%. The temperature where the magnitude of loss was to be evaluated was set to 191 °C, since this is the start of the degradation of the ASC inker [29]. The decomposition of the framework that follows includes the loss of ASC linkers, so the magnitude of the weight loss will be reduced when defects are present. However, it is impossible to distinguish between a missing linker and missing node defects using TGA alone because both types of defects result in bulk linker deficiency of the framework [27].

**Figure 5:**
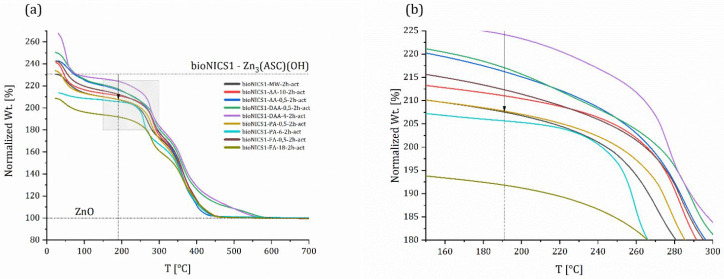
Comparison of thermogravimetric curves of selected, activated bioNICS1 materials crystalized under microwave heating for 2 h (**a**). Grey rectangle is magnified and presented in (**b**), where the line and arrow are set to the 191 °C marking the start of degradation of the linker.

When comparing pristine materials, it is observed that synthesis in MW alone was not sufficient to cause defects (Figure S17); however, when pristine materials were compared with modified ones according to the selected synthesis method and time (Figures S18 and S19), there were some observable changes. Firstly, the synthesis in a conventional oven (3 days) does not produce defects, which was expected. Synthesis time is long and with the addition of smaller amount of acids, crystallization slows down even further, giving crystals time to form more properly. There are some observable changes in the case of three samples with one day synthesis, but after analyzing the wt.% of inorganic residues (presented in Table S1) the results do not point towards a linker deficiency. In the case of MW synthesis, the combination of fast crystallization and addition of acids (=modulators) seems to produce some defects in the structure. Although there are smaller changes in the case of acetic acid in 1 h synthesis (Figure S19a and a.1), two major changes are visible for samples synthesized with formic acid (18 mol) and propionic acid (6 mol) in 1 and 2 h synthesis (Figure 5).

To shed some light on the types of point defects, we examined three samples (pristine/non-modified material—bioNICS1-MW-2h and two modified ones—bioNICS1-FA-18-2h and bioNICS1-PA-6-2h). Sample bioNICS1-FA-18-2h stands out for having the lowest magnitude of weight loss, the highest inorganic residue, but lower BET surface in comparison to non-modified sample. With bioNICS1-PA-6-2h we do observe a lower magnitude of loss in the TG curve, but no significant change in the amount of inorganic residue; we do, however, observe a significant increase in the BET surface.

**Figure 6:**
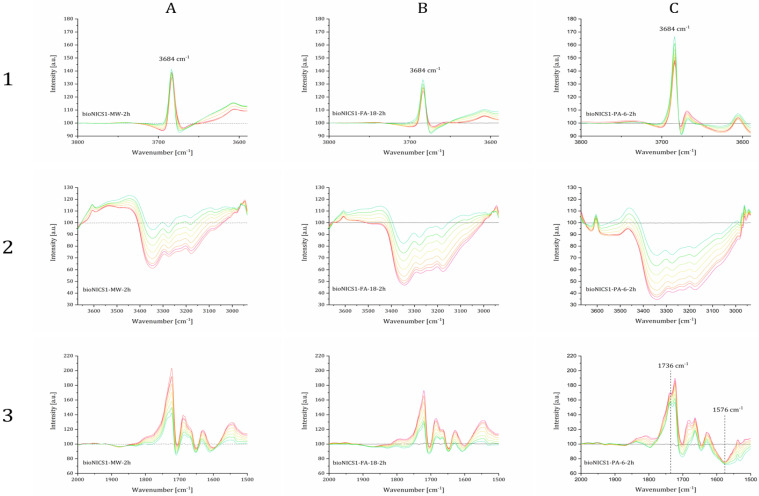
DRIFTS spectra of the specified samples purged with 10 % NH_3_ in Ar measured from room temperature (red lines) to 150 °C (green lines) shown in three wavenumber regions. Black lines represent the baseline of the activated materials. Represented samples are bioNICS1-MW-2h (**A**), bioNICS1-FA-18-2h (**B**) and bioNICS1-PA-6-2h (**C**).

Point defects, whether they are in the form of missing linker or missing nodes, generally correlate to the overall structure acidity. The presence of acid sites was, therefore, evaluated by the DRIFTS measurements on the selected samples. The difference in DRIFTS spectra after purging the samples with 10 % NH_3_ in argon and subsequent stepwise heating up to 150 °C are shown in transmittance on Figure 6.

**Figure 7:**
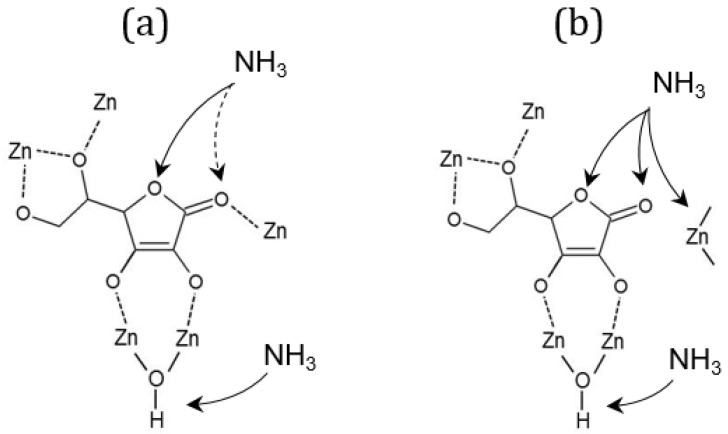
Scheme of the proposed acid binding sites for ammonia in (**a**) bioNICS1-FA-18-2h and (**b**) bioNICS1-PA-6-2h.

Relevant changes in spectra during the ammonia adsorption and temperature-programmed desorption process can be observed in three wavenumber regions. A positive sharp and intensive adsorption band at 3684 cm^−1^, that occurs in all cases (Figure 6: 1A–C), indicates the absence of O-H stretches due to the interaction of the Brønsted µ^3^-hydroxyl group with NH^4+^ species [30]. An additional broader and weaker band loss at 3670 cm^−1^ can be observed only in the spectra of bioNICS1-PA-6-2h, indicating the presence of another type of Zn-OH species which apparently does not exist in pristine and formic acid-modified bioNICS1 materials. Coordination of ammonia to the structure framework acid sites is confirmed by the presence of broad adsorption bands between 3500 and 3000 cm^−1^ due to the symmetric and asymmetric N-H stretching [31] (Figure 6: 2A–C). Band intensities are decreasing with the temperature due to the gradual desorption of ammonia upon heating. Integration of the corresponding band region reveals the relative amounts of adsorbed ammonia within the bioNICS1 materials. While bioNICS1-FA-18-2h shows only slight increment of the ammonia adsorption amount in comparison to the pristine bioNICS1, this enhancement is much more profound in the case of bioNICS1-PA-6-2h (Figure S20). Indicative differences in band occurrences upon ammonia adsorption can be observed in the region between 1800 and 1500 cm^−1^ as well (Figure 6: 3A–C). All spectra exhibit an intensive positive band at 1721 cm^−1^ which can be assigned to NH_3_ binding to the furyl oxygen site. This band is accompanied by the weaker shoulder at 1734 cm^−1^ in the spectra of pristine and formic acid-modified bioNICS1 and it appears in significantly higher intensity in the case of bioNICS1-PA-6-2h. The band is characteristic for stretching vibrations of C=O of the lactone ring [32]. More profound consumption of the carbonyl group upon NH_3_ adsorption in bioNICS1-PA-6-2h structure implies its higher activity as a possible result of a partial lack of coordination to the Zn(II) center. The presence of open metal sites in propionic acid-modified material is additionally proved by the occurrence of an adsorption band at 1567 cm^−1^, assigned to Zn-N stretching, due to the formation of Zn-NH_2_ moieties [33,34]. DRIFTS confirmed higher diversity of ammonia-to-framework interactions in the case of bioNICS-PA-6-2h (Figure 7b), with respect to the pristine and formic acid-modified materials (Figure 7a) which implies that propionic acid acts as a modulator, with the ability to provide a missing node defect into the bioNICS1 structure, thus increasing the overall number of acid sites in the material.

**Figure 8:**
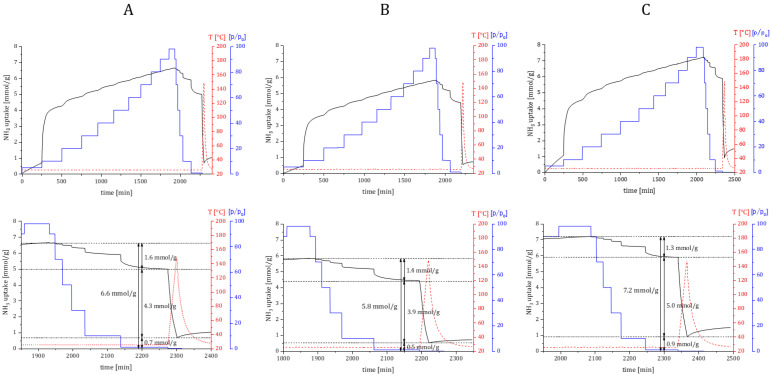
Ammonia sorption kinetics profile of selected materials ((**A**) bioNICS1-MW-2h; (**B**) bioNICS1-FA-18-2h; (**C**) bioNICS1-PA-6-2h) and corresponding inserts (below) showing physiosorbed and chemisorbed fractions of adsorbed ammonia, together with remaining NH3 derivative species. Ammonia dosing pressure—blue line, heating temperature—red line.

Ammonia DRIFTS experiments provided another valuable piece of information about the existence of different types of acid sites on bioNICS1 framework structures. Furthermore, quantitative evaluation of acidity was performed by the dynamic adsorption/desorption isothermal measurements, where activated materials were gradually loaded in 10% NH_3_/Ar flow, followed by pressure swing and subsequent temperature-programmed desorption with heating up to 150 °C (at temperatures above this the integrity of the bioNICS1 framework could not be guaranteed). Mass difference between the total uptake and uptake after pressure swing desorption corresponds the removal of weakly bonded or physiosorbed NH_3_, whereas the remaining amount is considered as chemisorbed NH_3_ which can be directly correlated to the amount of acid sites (Figure 8).

Unmodified bioNICS1 possess acid sites with a total amount of 4.3 mmol/g. The total acidity is somewhat decreased to 3.9 mmol/g in the case of bioNICS1-FA-18-2h, which can be explained by the lower accessibility of sorption sites due to the lower specific surface area. On the other hand, bioNICS1-PA-6-2h exhibits higher acidity (5.0 mmol/g) than the pristine material. These results are in agreement with the predicament made with analysis of TG curves (smallest magnitude of weight loss and highest inorganic residue) and DRIFTS experiments; the involvement of modulators was also confirmed with liquid NMR (Figure S21). The generation of defects in the bioNICS1-FA-18-2h sample cannot be so easily explained, since the decrease in the BET surface is also related to the formation of another phase (see Supplementary Figures S22 and S23). The results do confirm that the existence of linker deficiency is a consequence of present missing node defects when using propionic acid as an additive; this causes not only the enhancement of the material’s acidity, but also generates additional microporous voids within the MOF framework (Figure S24) contributing to higher specific area. The resilience of the structure after exposure to NH_3_ was checked and confirmed with PXRD (Figure S25).

## 3. Materials and Methods

### 3.1. Synthetic Approaches

Starting reaction mixture:

BioNICS-1 was prepared using EtOH as a solvent with Zn/ASC ratio of 2:1. Typically 0.752 g of zinc(II) acetatedehydrate (Sigma Aldrich, St. Louis, MO, USA) and 0.3 g of L-ascorbic acid (Sigma-Aldrich, St. Louis, MO, USA) were added to 9.97 ml of ethanol (Sigma-Aldrich, St. Louis, MO, USA).

Modulation addition:

After mixing the reaction mixture described above, chosen modulators were added in different molar ratios ranging from 0.1 mol to 22 mol; setting the reaction ratio to Zn/ASC/EtOH/modulator = 2:1:100: 0.1–22

Used modulators:

Formic acid—FA/fa (Sigma-Aldrich, St. Louis, MO, USA, 95%), acetic acid—AA/aa (Merck, Rahway, NJ, USA, 100%) propionic acid—PA/pa (Sigma-Aldrich, St. Louis, MO, USA, 99.5%) and dichloroacetic acid—DAA/daa (Sigma-Aldrich, St. Louis, MO, USA, 98.0%).

Conventional heating:

The reaction mixture was heated in a conventional heating oven at 120 °C for 1 and 3 days in Teflon-lined stainless-steel autoclaves. Obtained samples were denoted as bioNICS1- concentration, type of additive (lower case letters), heating time (days); for example: bioNICS1-6(pa)-1.

Microwave assisted heating:

The reaction mixture was heated in a microwave oven (600 W) at 120 °C for 1 and 2 h in Teflon-lined autoclaves. Obtained samples were denoted as bioNICS1- type of additive (UPPER CASE letters), concentration, heating time (h); for example: bioNICS1-DAA-4-1h.

Activation procedure:

This followed a simple “wash”, which was conducted in a round bottom flask with reflux cooling on an oil bath in absolute EtOH, at 60 °C, under constant stirring for approximately 12 h. Following the filtration, the product was dried in a vacuum oven at 100 °C for 3 h.

### 3.2. Characterization Methods

X-ray powder diffraction data of the samples were collected on a PANalytical X’Pert PRO high-resolution diffractometer (Malvern Panalytical, Almelo, The Netherlands) with CuKa radiation (λ = 1.5406 Å) in the range from 5 to 60° (2θ) with the step of 0.034° per 100 s using fully opened 100 channel X’Celerator detector. For the purposes of Rietveld refinement of the crystal structure model, the XRD powder data were collected on the same equipment using a transmission mode in the range from 5 to 35° 2Θ with the step of 0.016°/300 s. The diffractograms were analyzed and the particle size calculated using the Sherrer equation with the HighScore Plus 4.9 program package (Malvern Panalytical B.V.).

Morphological properties and size estimation of the samples was observed by scanning electron microscopy measurements (SEM) on Zeiss Supra™ 3VP field-emission gun (FEG) microscope (Carl Zeiss AG, Oberkochen, Germany). Elemental analysis was performed by energy dispersive X-ray analysis (EDAX) with an INCA Energy system attached to the above described microscope and by Perkin Elmer 2400 Series II CHNS analyser (Perkin Elmer, Waltham, MA, USA).

The thermal analysis (TG/DTG) was performed on a Q5000 IR thermogravimeter (TA Instruments, Inc., New Castle, DA, USA). The measurements were carried out in air flow of 10 ml/min, by heating samples from 25 °C to 700 °C at a rate of 10 °C/min. The temperature-programmed X-ray powder diffraction pattern of samples was recorded also on the PANalyticalX’Pert PRO diffractometer, additionally equipped with a high temperature sample cell, from room temperature to 500 °C in steps of 50 °C in static air.

N_2_ sorption isotherms measurements were performed on Quantachrome AUTOSORB iQ3 (Anton Paar, Graz, Austria). The specific surface areas were determined by Brunauer–Emmett–Teller (BET) method based on the N_2_ sorption isotherms measured at 77 K in p/p0 relative pressure range between 4 × 10^−2^ and 6 × 10^−3^, selected according to Roquerol plots. Before the measurement, samples were activated under vacuum at 150 °C for 15 h. Pore size distribution analysis (PSD) was performed using the NLDFT procedure based on the adsorption data.

Dynamic light scattering (DLS) measurements were carried out on Zetasizer Nano ZS (Malvern Instruments, Almelo, The Netherlands). Prior to the measurements, the powdered sample was dispersed in water by sonification in an ultrasonic bath for 10 min. The dispersion was filtered through a 0.8µm filter attached to a syringe and transferred into a polystyrene cuvette for the measurement. Data evaluation was performed using Malvern Zetasizer software. The parameters chosen for the evaluation were those for ZnO material (refractive index of 2.003 and adsorption of 0.01). The number of particles with a mean diameter were calculated.

Zeta potential measurements were carried out on Zetasizer Nano ZS Malvern Instruments. Prior to the measurements, the powdered sample was dispersed in water by sonification in an ultrasonic bath for 4 min with approximately 20% power. The dispersion was filtered through a 0.8 µm filter attached to a syringe and transferred into a polystyrene cuvette for the measurement. pH values of the measured suspensions (pH 2–9) were adjusted with 0.1 M HCl and 0.1 M NaOH solutions.

For ammonia adsorption experiments, the activated samples were heated with a ramp of 10 °C min^−1^ to 150 °C in Ar flow (purity 5.0) and held at this temperature overnight. After pretreatment, the FTIR spectrum of activated materials was collected and used as a background. The samples were then purged with 10% NH_3_ in Ar (purity 5.0) for 30 min at room temperature and then gradually heated with the ramp of 10 °C min^−1^ to 150 °C. FITR spectra were recorded for every 20 °C. DRIFTS analysis was performed using 10 mg of finely powdered catalyst, loaded into a porous ceramic cup and placed in the DiffusIR cell (PIKE Technologies, Fitchburg, WI, USA) attached to a FTIR spectrometer (Perkin Elmer, model Frontier, Waltham, MA, USA). The background was recorded with the powdered catalyst using an LN2 cooled MCT detector. Spectra were recorded between 400 and 4400 cm^−1^, 32 accumulations per scan and spectral resolution of 4 cm^−1^.

Acid sites were quantitatively evaluated using ammonia adsorption/adsorption experiments on a dynamic vapor sorption analyzer (DVS, Surface Measurement Systems Ltd., London, UK). Prior the measurements, the samples were outgassed at 150 °C for 12 h. Ammonia dynamic adsorption was performed on activated materials using 10% NH_3_ in argon flow of 5 ml/min, gradually increasing pressure from vacuum to 1 bar with the step of 100 mbar measuring equilibrium mass gain for each pressure step. The desorption process, using steps of 200 mbars, was followed by heating up to 150 °C with a ramp of 10 °C/min.

## 4. Conclusions

Zinc ascorbate MOF structure with permanent microporosity (bioNICS-1) and biocompatible constituents has an inherent potential in the biomedical field as a drug delivery system. The tuning of textural properties and controlled generation of acid sites was achieved by utilizing simple monocarboxylic acids as a way of manipulating a compositional parameter of synthesis. We confirmed that with the right choice of an additive and its concentration, we can control the size of the crystallites according to the seesaw model, and in the case of dichloroacetic acid also surface morphology. The general conclusion is that in the case of conventional heating, additives act as surface capping agents and blocking agents, whilst in the case of microwave synthesis, their role changes to modulators. This was proven specifically in the case of propionic acid, where missing node defects were observed resulting in generation of additional acid sites. With this study, tunability of the bioNICS1 framework was proven, increasing its value as a candidate for drug delivery systems. 

## Figures and Tables

**Table 1:** pKa and molecular size values for the chosen additives.

Additive	pKa	Molecular Size (Å)
formic acid (fa, FA)	3.75	3.3
acetic acid (aa, AA)	4.76	3.8
dichloroacetic acid (daa, DAA)	1.35	4.4
propionic acid (pa, PA)	4.88	4.8

## Data Availability

The data presented in this study are available on request from the corresponding authors.

## Supplementary Materials

The following supporting information can be downloaded at: https://www.mdpi.com/article/10.3390/molecules28010253/s1, Figure S1: SEM micrographs of bioNICS1 with acetic acid as an additive. Figure S2: SEM micrographs of bioNICS1 with dichloroacetic acid as an additive. Figure S3: SEM micrographs of bioNICS1 with formic acid as an additive. Figure S4: SEM micrographs of bioNICS1 with propionic acid as an additive. Figure S5: PXRD of pristine bioNICS1 obtained from different synthesis pathways. Figure S6: PXRD of all samples utilizing acetic acid as an additive. Figure S7: PXRD of all samples utilizing dichloroacetic acid as an additive. Figure S8: PXRD of all samples utilizing formic acid as an additive. Figure S9: PXRD of all samples utilizing propionic acid as an additive. Figure S10: Calculated size of crystal domains plotted against molar addition of corresponding acid. Figure S11: Starting point is set to the smallest size. Figure S12: (a) N_2_ isotherms of the selected bioNICS1 products with the addition of different acids under conventional heating conditions. (b) Pore size distribution of selected bioNICS1 products with the addition of different acids under conventional heating. Figure S13: (a) N_2_ isotherms of the selected bioNICS1 products with the addition of different acids under conventional heating conditions. (b) Pore size distribution of selected bioNICS1 products with the addition of different acids under conventional heating. Figure S14: (a) N_2_ isotherms of the selected bioNICS1 products with the addition of different acids under microwave heating conditions. (b) Pore size distribution of selected bioNICS1 products with the addition of different acids under conventional heating. Figure S15: (a) N_2_ isotherms of the selected bioNICS1 products with the addition of different acids under microwave heating conditions. (b) Pore size distribution of selected bioNICS1 products with the addition of different acids under conventional heating. Figure S16: Zeta potential of selected bioNICS1 materials dependence on pH of aqueous medium. Figure S17: Comparison of thermogravimetric curves of pristine (and activated) bioNICS1 materials differing in synthesis conditions. Figure S18: Comparison of thermogravimetric curves of selected, activated bioNICS1 materials crystalized under conventional heating. Figure S19: Comparison of thermogravimetric curves of selected, activated bioNICS1 materials crystalized under microwave heating. Figure S20: Integrated area of bands between 3450–3000 cm^−1^. Figure S21: ^1^H NMR spectra of the selected bioNICS-1 samples degraded in DCl solution. Figure S22: Comparison of thermogravimetric curves of bioNICS1-FA-18-2h material before (syn) and after (act) activation process. Figure S23: PXRD diffraction pattern of synthesized and activated bioNICS1-FA-18-2h. Figure S24: Comparison of pore size distribution of selected samples. Figure S25: Comparison of PXRD diffractograms confirming the original bioNICS1 structure of modulated and unmodulated before and after FTIR and DVS experiments.

## Sample Availability

Samples of the compounds are not available from the authors.
