# Supplementary material for: Tuning Size and Properties of Zinc Ascorbate Metal-Organic Framework via Acid Modulation
Source: Molecules. 2022 Dec 28;28(1):253. doi: 10.3390/molecules28010253 (PMC9822160; doi:10.3390/molecules28010253)
Supplement: Supplementary file 1 [file molecules-28-00253-s001.zip › molecules-2097098-supplementary.pdf]

Supporting information

Tuning size and properties of zinc ascorbate metal-organic framework via acid modulation

Tia Kristian Tajnšek [1, 2], Nataša Zabukovec Logar [1,3] and Matjaž Mazaj [1,*]

[1] National Institute of Chemistry, Hajdrihova 19, 1000 Ljubljana, Slovenia

[2] Faculty of Inorganic Chemistry and Technology, University of Ljubljana, Večna pot 113, 1000 Ljubljana, Slovenia

[3] University of Nova Gorica, Vipavska 13, 5000 Nova Gorica, Slovenia

Corresponding author: Matjaž Mazaj, matjaz.mazaj@ki.si

Contents

1. SEM 3
2. PXRD difractograms 7
3. Size analysis........ 12
4. N_2 adsorption and pore size distribution 13
5. Zeta potential........ 15
6. Thermogravimetric analysis........ 15
7. Study of defects 19

1. SEM

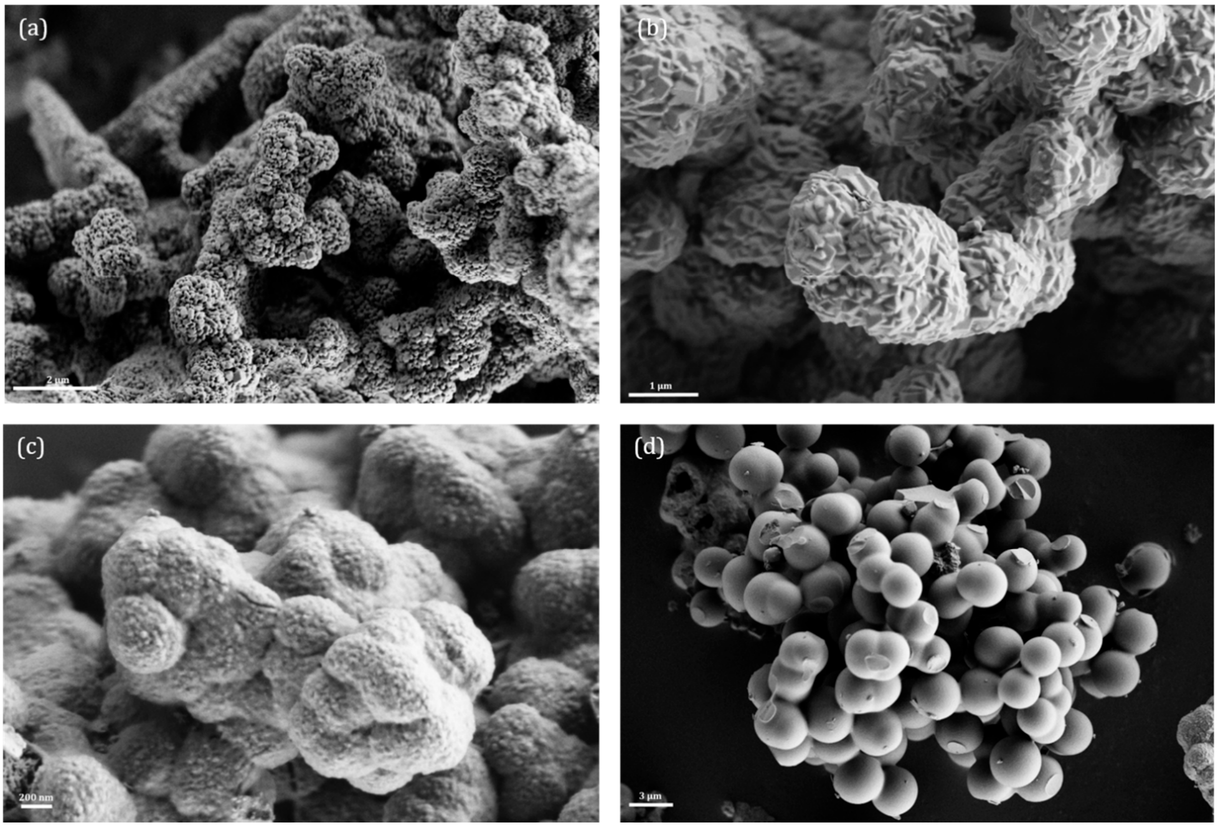

Figure S1: SEM micrographs of bioNICS1 with acetic acid as an additive. (a) and (b) represent samples after microwave synthesis with low and high molar addition of the acid, bioNICS1-AA-4-1h and bioNICS1-AA-10-2h, respectively. (c) and (d) represent samples after conventional heating synthesis with low and high molar addition of the acid, bioNICS1-0,5(aa)-1 and bioNICS1-16(aa)-3, respectively.

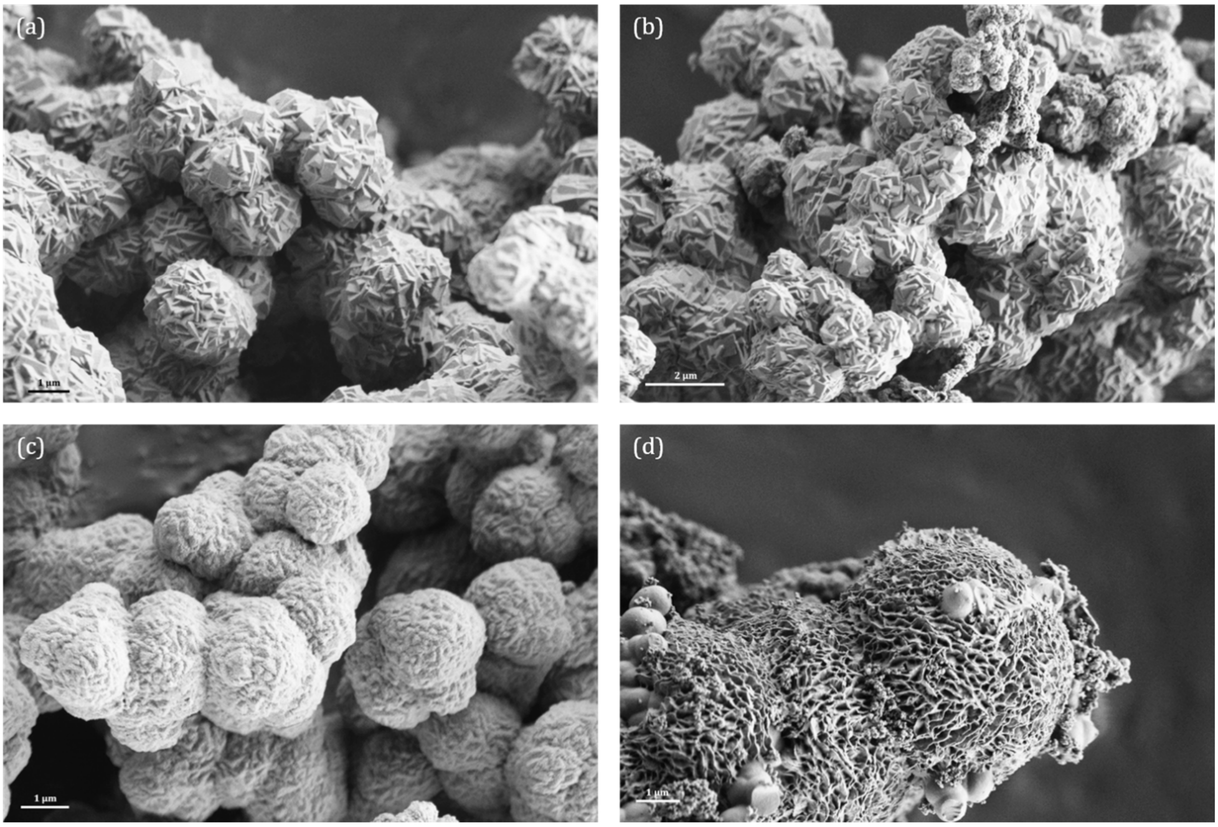

Figure S2: SEM micrographs of bioNICS1 with dichloroacetic acid as an additive. (a) and (b) represent samples after microwave synthesis with low and high molar addition of the acid, bioNICS1-DAA-1,5-1h and bioNICS1-DAA-4-2h respectively. (c) and (d) represent samples after conventional heating synthesis with low and high molar addition of the acid, bioNICS1-1,5(daa)-1 and bioNICS1-4(daa)-1, respectively.

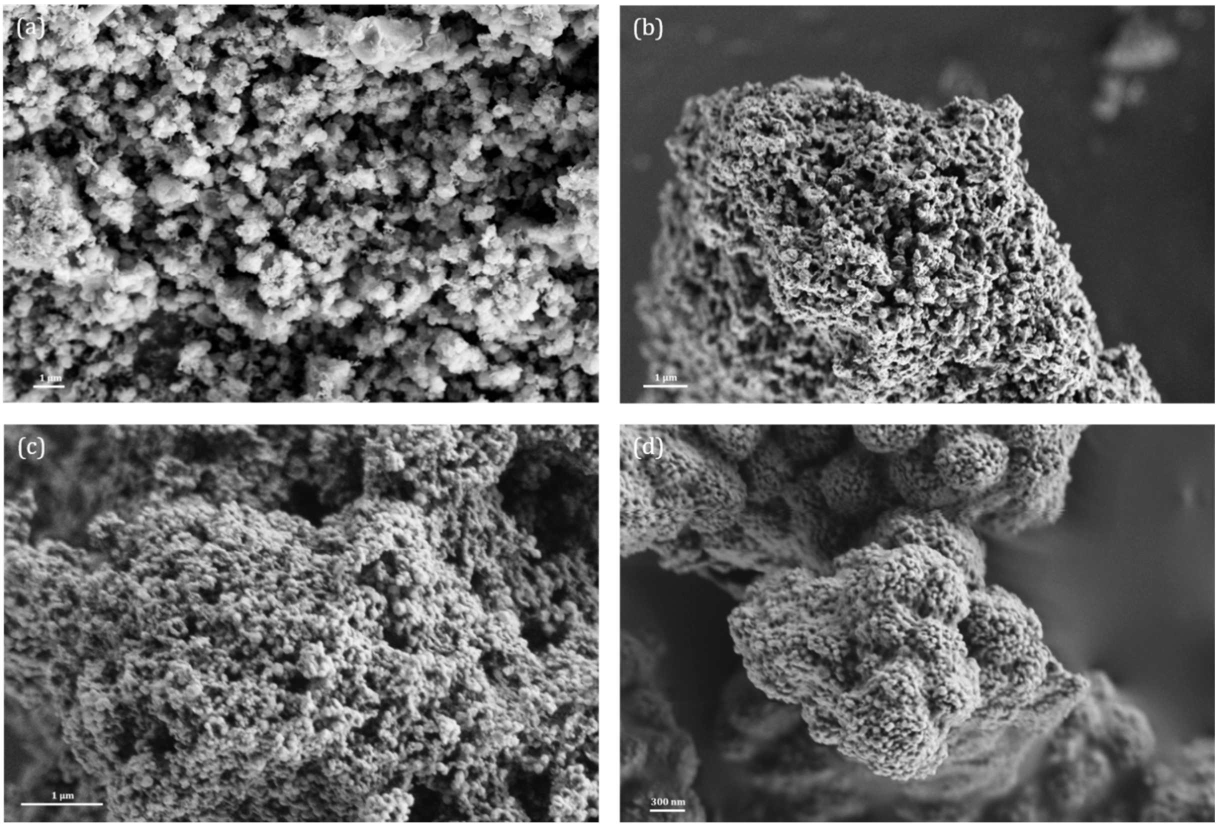

Figure S3: SEM micrographs of bioNICS1 with formic acid as an additive. (a) and (b) represent samples after microwave synthesis with low and high molar addition of the acid, bioNICS1-FA-0,5-1h and bioNICS1-FA-18-2h respectively. (c) and (d) represent samples after conventional heating synthesis with low and high molar addition of the acid, bioNICS1-1(fa)-1 and bioNICS1-6(fa)-3, respectively.

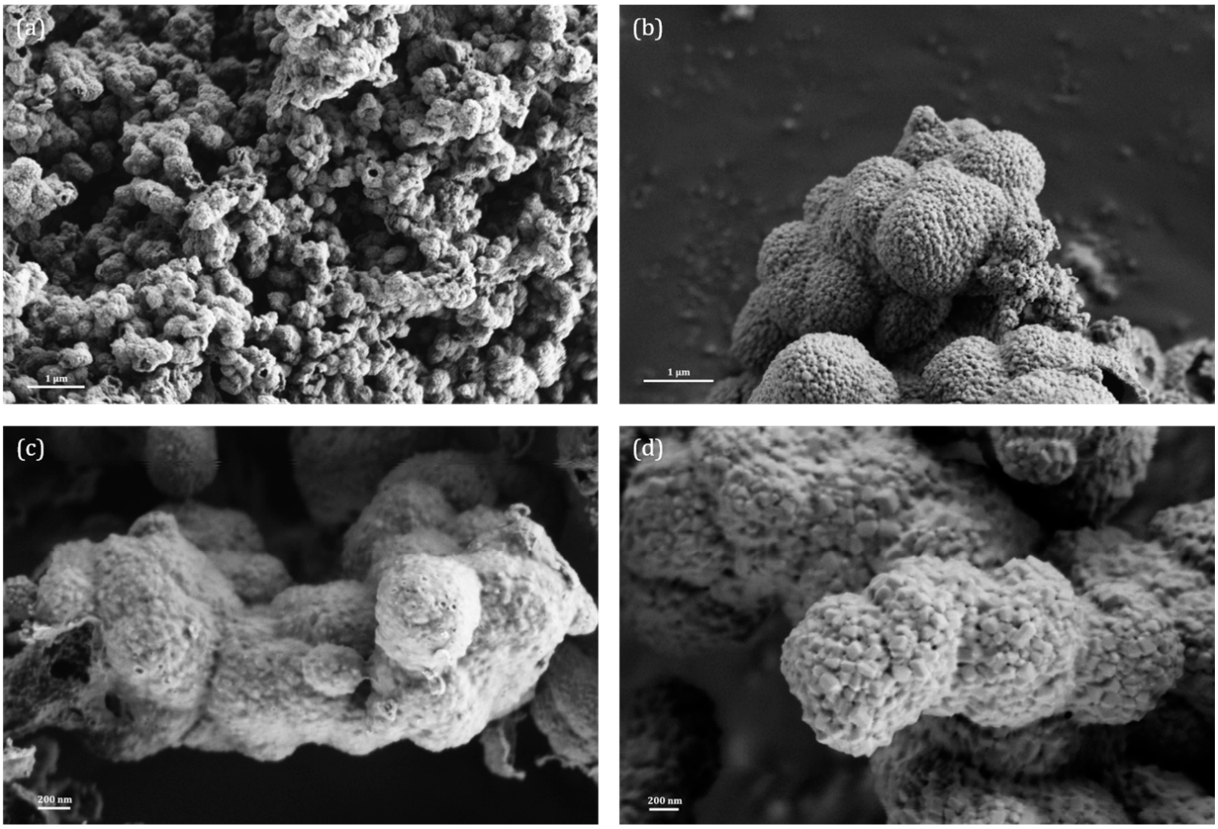

Figure S4: SEM micrographs of bioNICS1 with propionic acid as an additive. (a) and (b) represent samples after microwave synthesis with low and high molar addition of the acid, bioNICS1-PA-0,5-1h and bioNICS1-PA-6-2h, respectively. (c) and (d) represent samples after conventional heating synthesis with low and high molar addition of the acid, bioNICS1-0,5(pa)-1 and bioNICS1-6(pa)-3, respectively.

2. PXRD difractograms

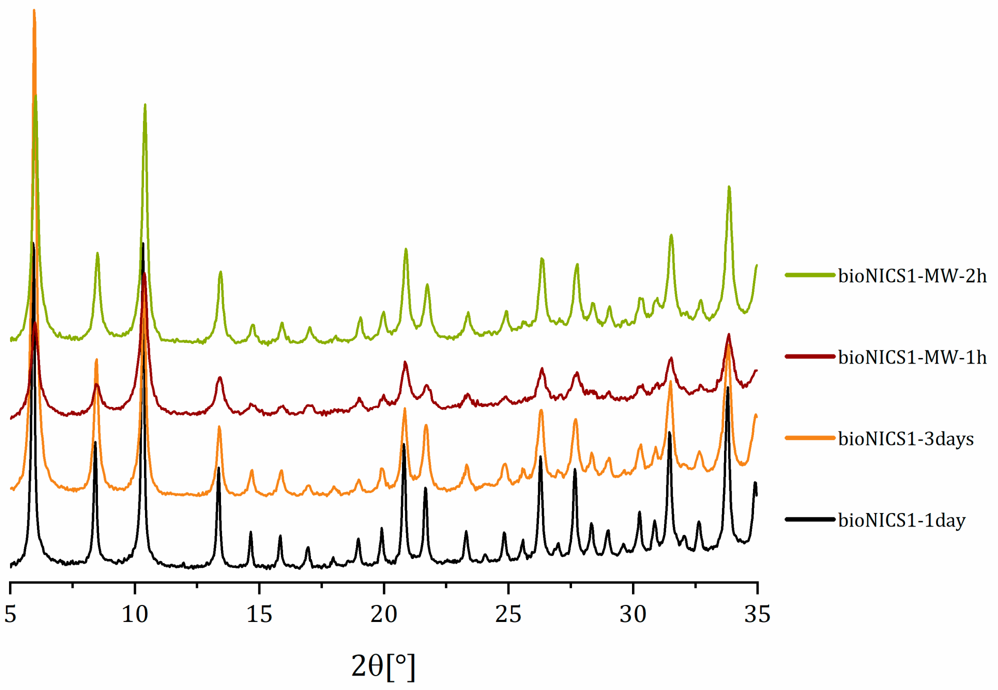

Figure S5: PXRD of pristine bioNICS1 obtained from different synthesis pathways.

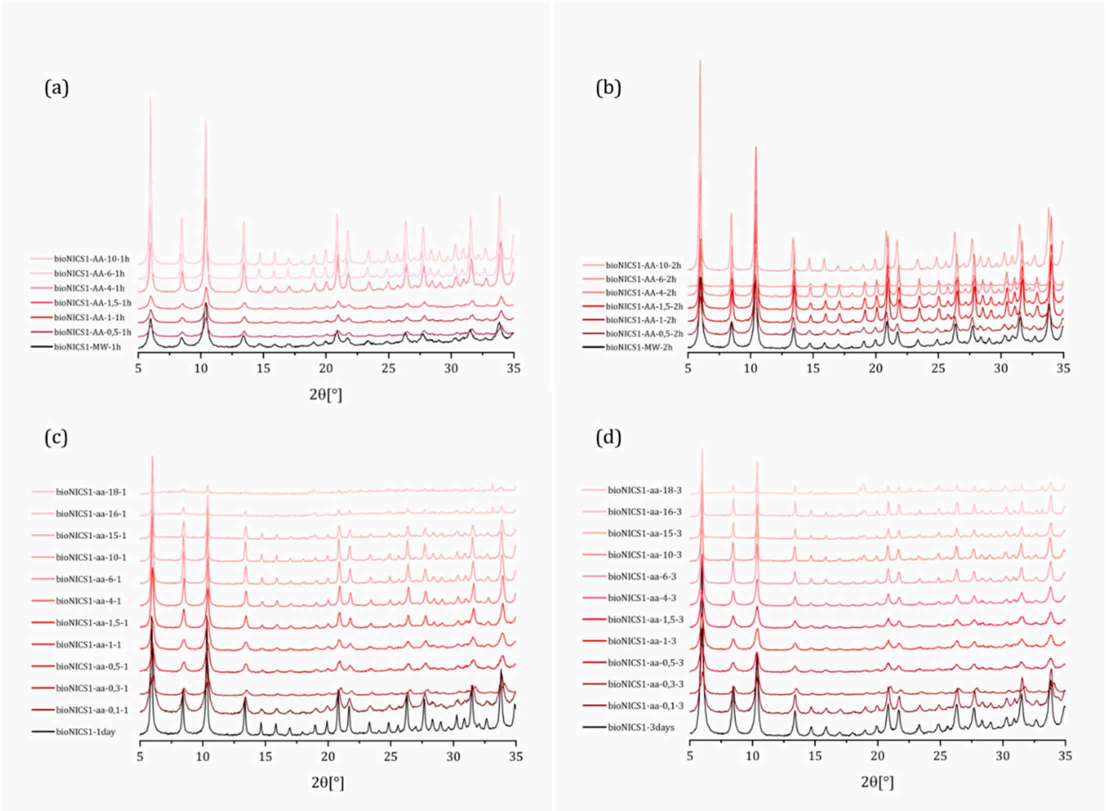

Figure S6: PXRD of all samples utilizing acetic acid as an additive. (a) and (b) represent the MW synthetic pathway; (c) and (d) represent synthesis in a conventional heating oven. Samples are denoted by framework name (bioNICS1), lowercase abbreviation (aa) for conventional synthesis or upper case (AA) for MW synthesis followed by a molar addition and time of the synthesis.

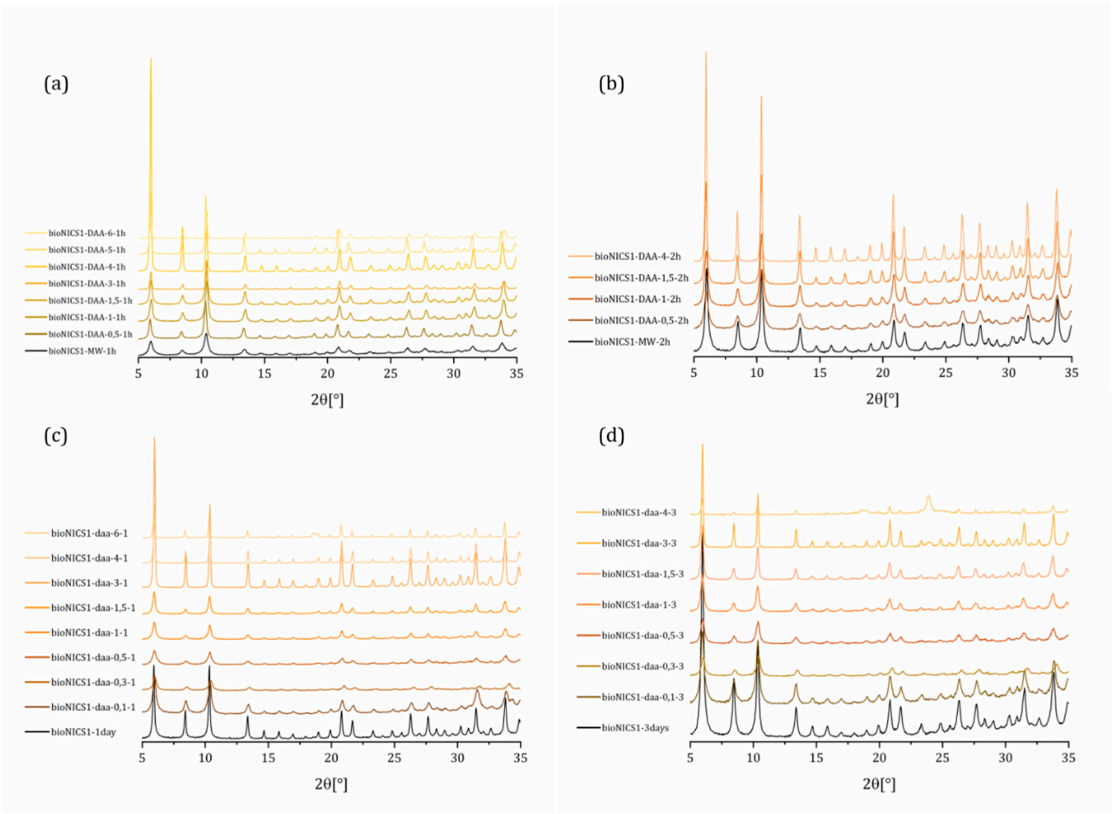

Figure S7: PXRD of all samples utilizing dichloroacetic acid as an additive. (a) and (b) represent the MW synthetic pathway; (c) and (d) represent synthesis in a conventional heating oven. Samples are denoted by framework name (bioNICS1), lowercase abbreviation (daa) for conventional synthesis or upper case (DAA) for MW synthesis followed by a molar addition and time of the synthesis.

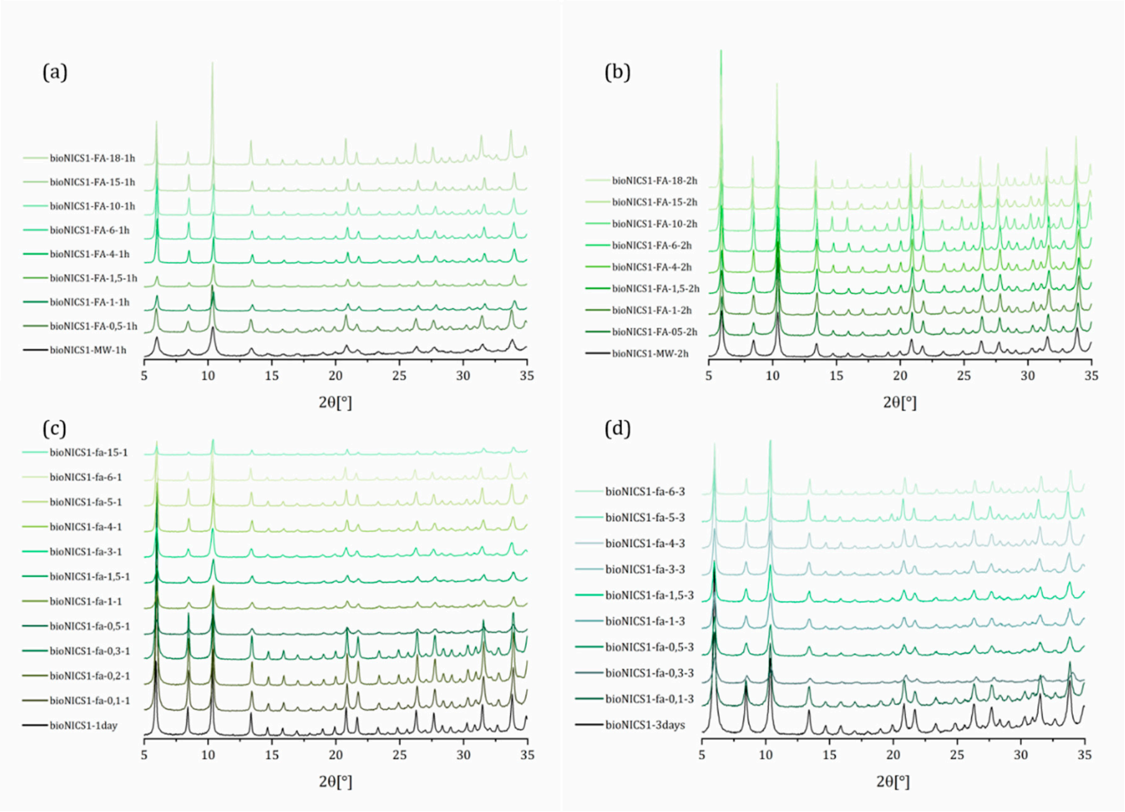

Figure S8 : PXRD of all samples utilizing formic acid as an additive. (a) and (b) represent the MW synthetic pathway; (c) and (d) represent synthesis in a conventional heating oven. Samples are denoted by framework name (bioNICS1), lowercase abbreviation (fa) for conventional synthesis or upper case (FA) for MW synthesis followed by a molar addition and time of the synthesis.

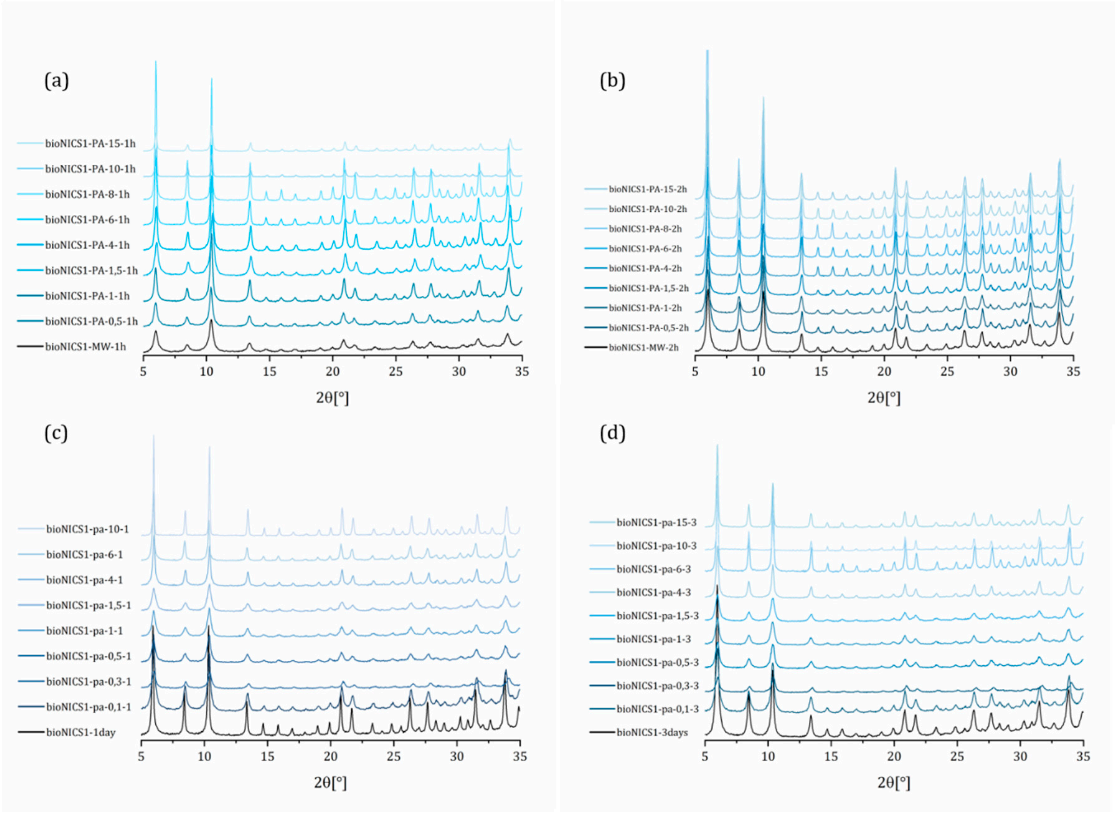

Figure S9: PXRD of all samples utilizing propionic acid as an additive. (a) and (b) represent the MW synthetic pathway; (c) and (d) represent synthesis in a conventional heating oven. Samples are denoted by framework name (bioNICS1), lowercase abbreviation (pa) for conventional synthesis or upper case (PA) for MW synthesis followed by a molar addition and time of the synthesis.

3. Size analysis

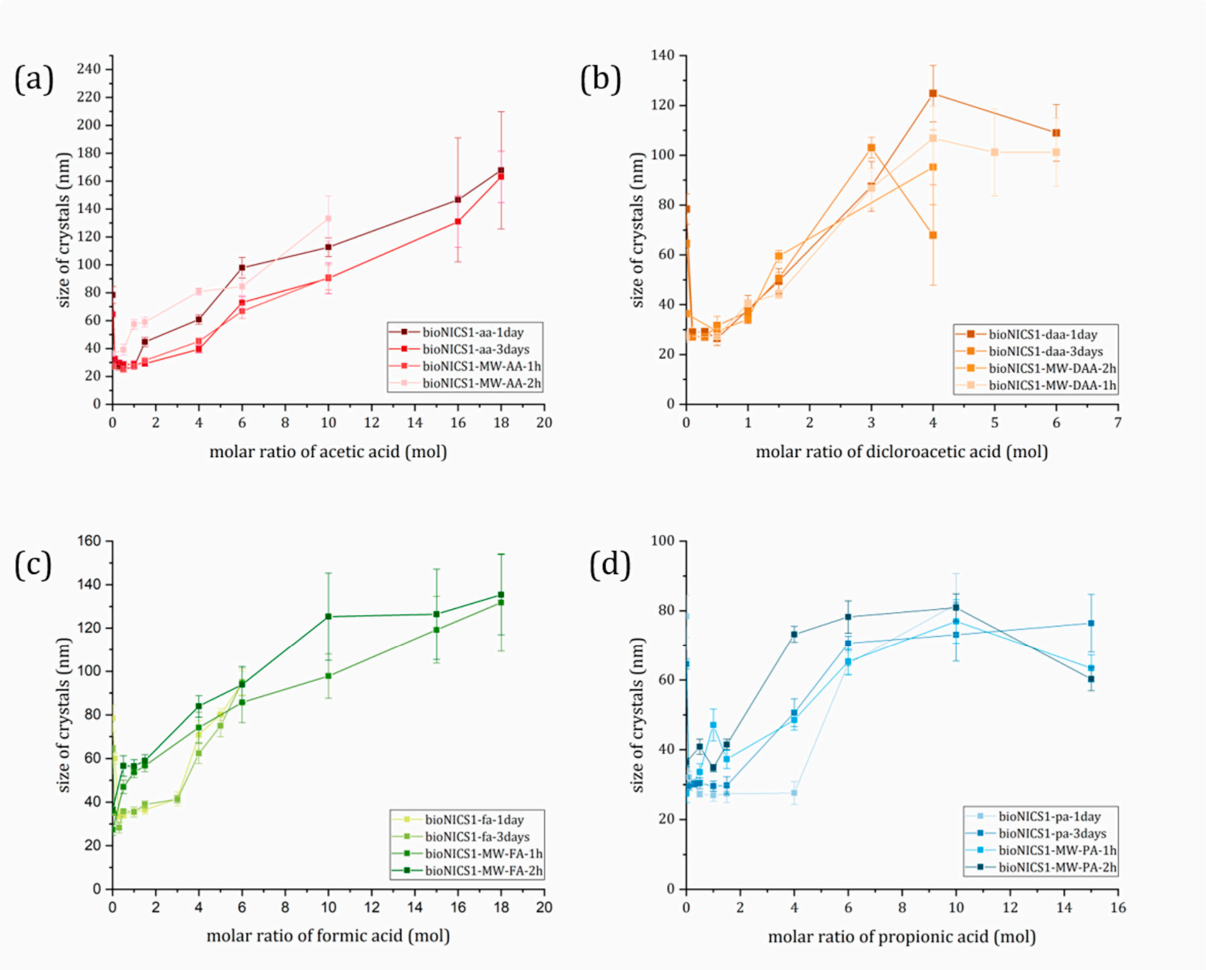

Figure S10: Calculated size of crystal domains plotted against molar addition of corresponding acid.

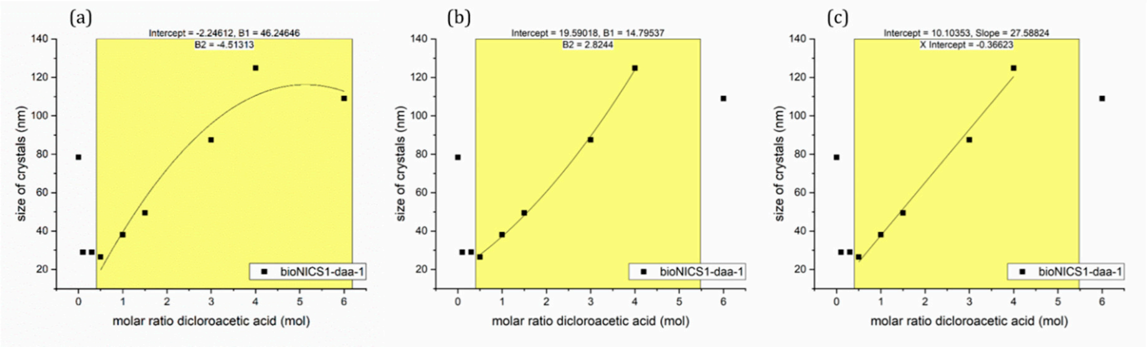

Figure S11: Starting point is set to the smallest size. If we chose the entire range to the right we do not necessary get the quadratic parabola (a). So the point on the far right is omitted and the curve that follows Regime II is set (b). Then we change the fit to linear one to calculate the slope of set range (c).

4. N_2 adsorption and pore size distribution

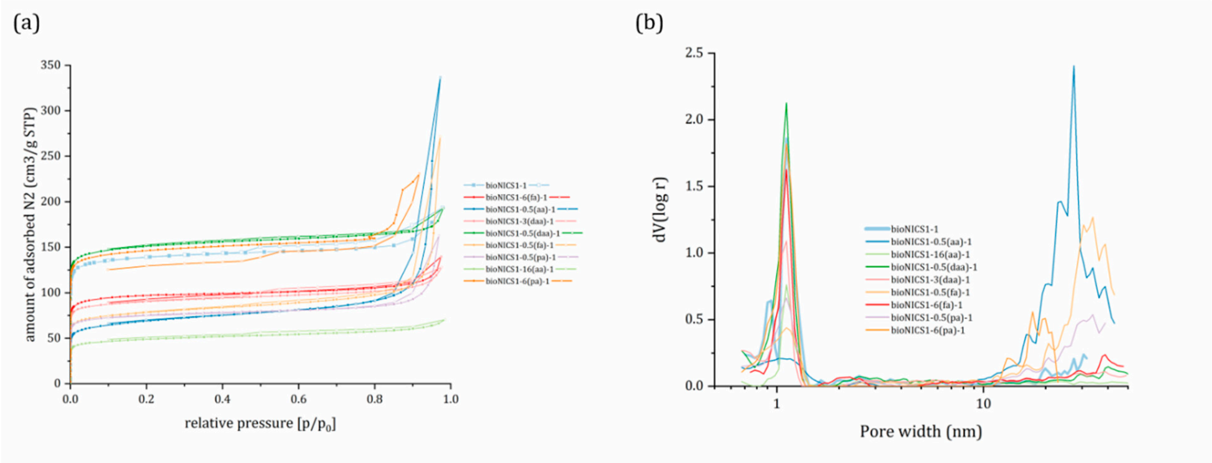

Figure S12: (a) N_2 isotherms of the selected bioNICS1 products with the addition of different acids under conventional heating conditions. Framework name (bioNICS1) - molar addition – (acid used) – 1 day synthesis. (b) Pore size distribution of selected bioNICS1 products with the addition of different acids under conventional heating. Samples are denoted by framework name (bioNICS1) - molar addition – (acid used) – 1 day synthesis.

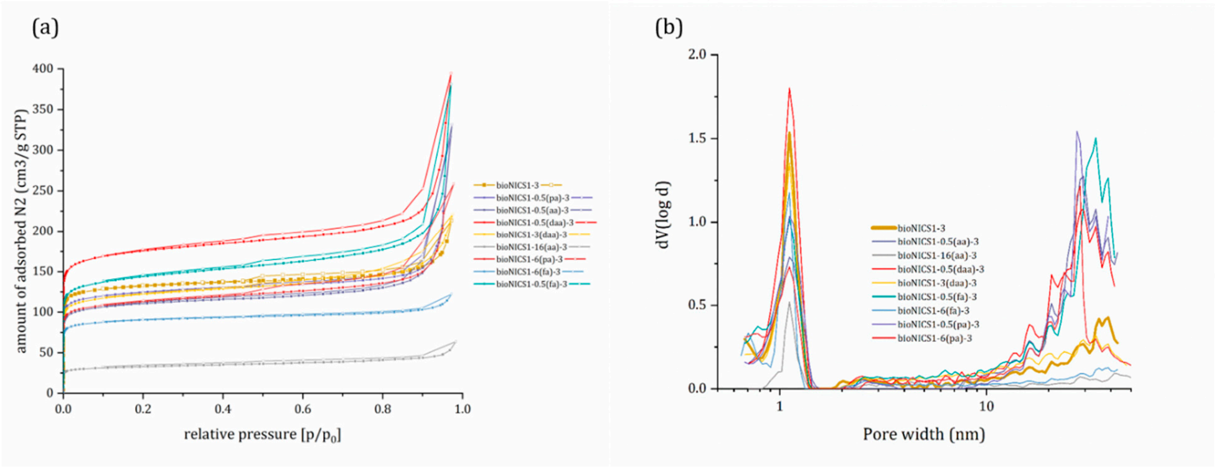

Figure S13: (a) N_2 isotherms of the selected bioNICS1 products with the addition of different acids under conventional heating conditions. Framework name (bioNICS1) - molar addition – (acid used) – 1 day synthesis. (b) Pore size distribution of selected bioNICS1 products with the addition of different acids under conventional heating. Samples are denoted by framework name (bioNICS1) - molar addition – (acid used) – 3 day synthesis.

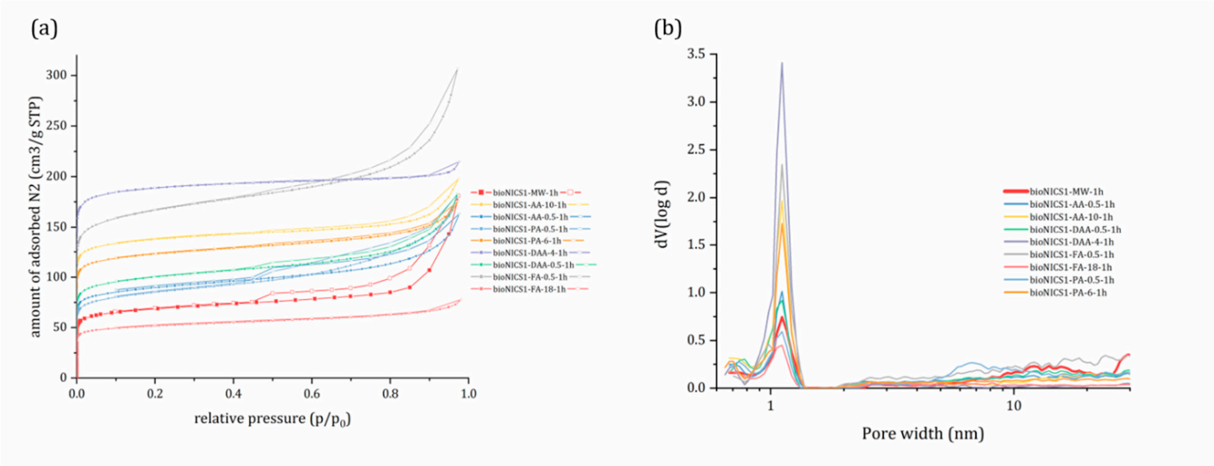

Figure S14: (a) N_2 isotherms of the selected bioNICS1 products with the addition of different acids under microwave heating conditions. Framework name (bioNICS1) - molar addition – (acid used) – 1 day synthesis. (b) Pore size distribution of selected bioNICS1 products with the addition of different acids under conventional heating. Samples are denoted by framework name (bioNICS1) - molar addition – (acid used) – 1h synthesis.

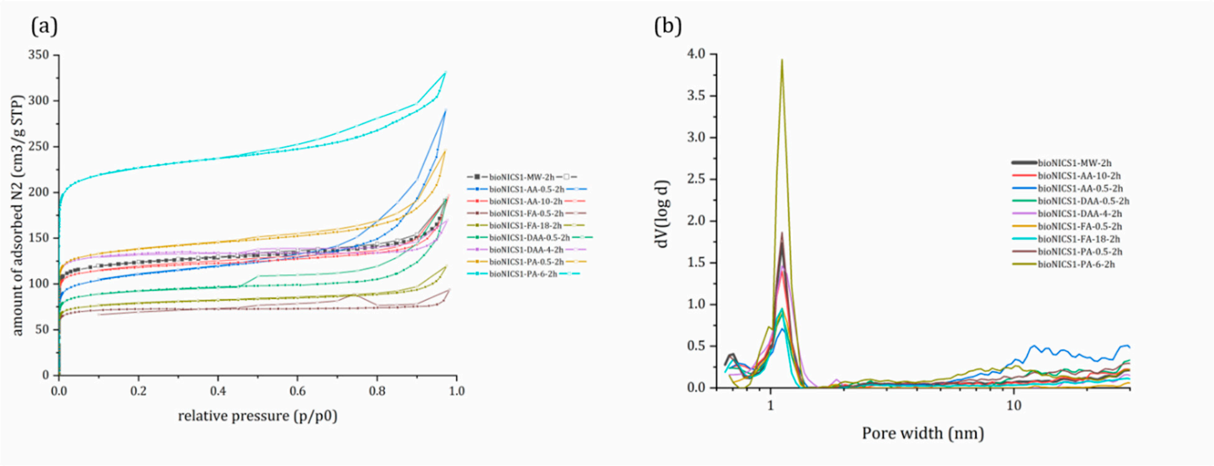

Figure S15: (a) N_2 isotherms of the selected bioNICS1 products with the addition of different acids under microwave heating conditions. Framework name (bioNICS1) - molar addition – (acid used) – 1 day synthesis. (b) Pore size distribution of selected bioNICS1 products with the addition of different acids under conventional heating. Samples are denoted by framework name (bioNICS1) - molar addition – (acid used) – 2h synthesis.

5. Zeta potential

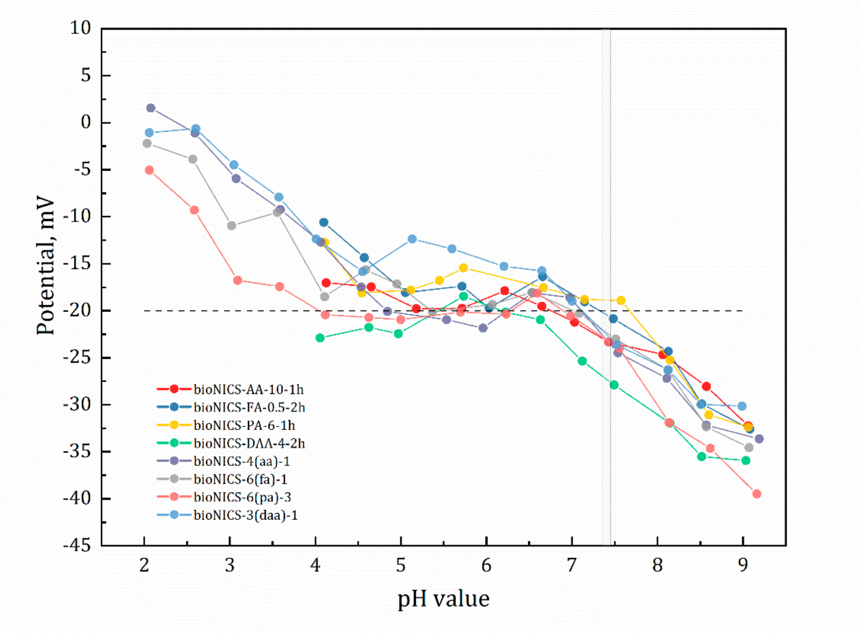

Figure S16: Zeta potential of selected bioNICS1 materials dependence on pH of aqueous medium.

6. Thermogravimetric analysis

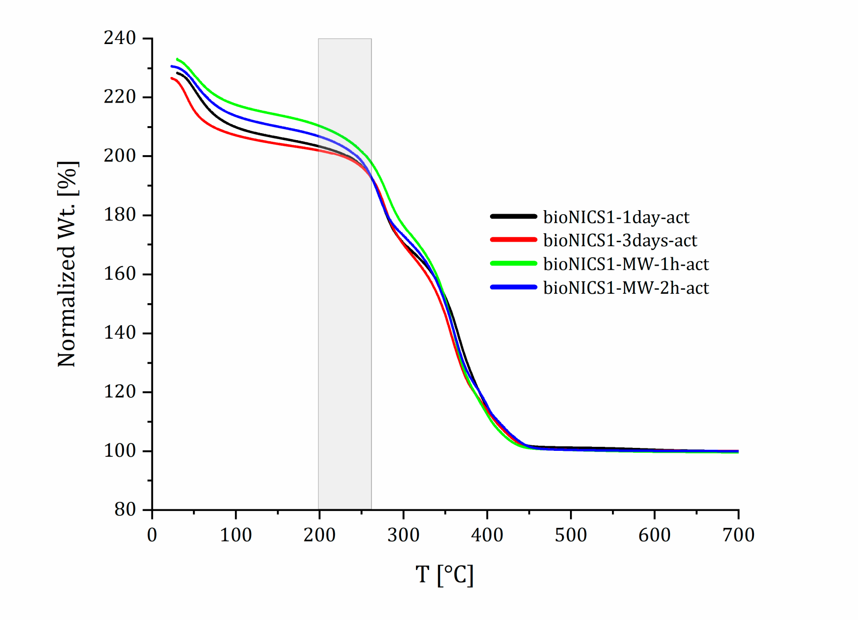

Figure S17: Comparison of thermogravimetric curves of pristine (and activated) bioNICS1 materials differing in synthesis conditions (conventional heating – 1 or 3 days and microwave heating (MW) – 1 or 2 h). The grey ribbon represents the temperature range of the first thermal degradation of linker – ascorbic acid.

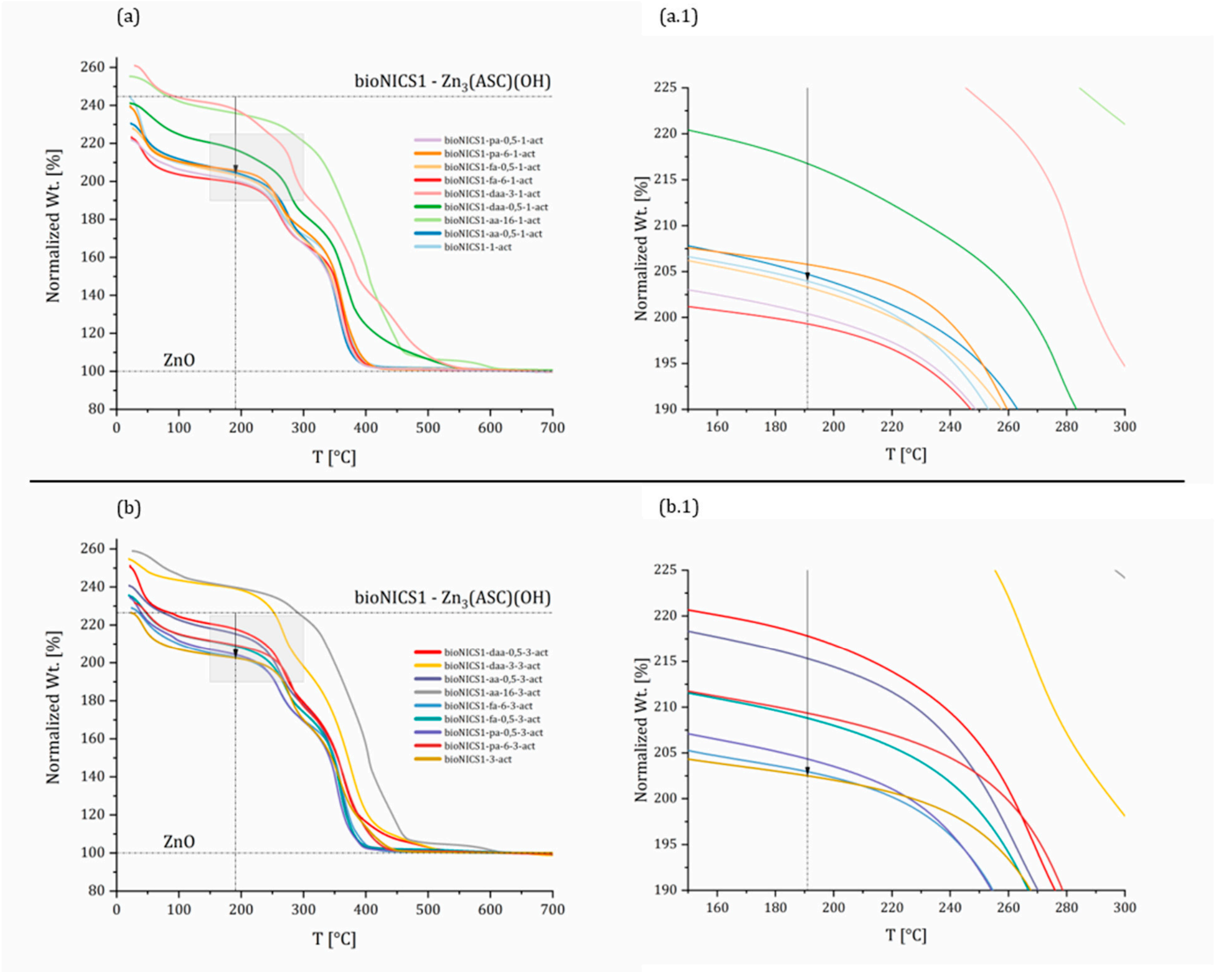

Figure S18: Comparison of thermogravimetric curves of selected, activated bioNICS1 materials crystalized under conventional heating for 1 day (a) or 3 days (b). Grey rectangle on (a) and (b) is magnified and presented in (a.1) and (b.1), where the line and arrow are set to the 191°C marking the start of degradation of the linker.

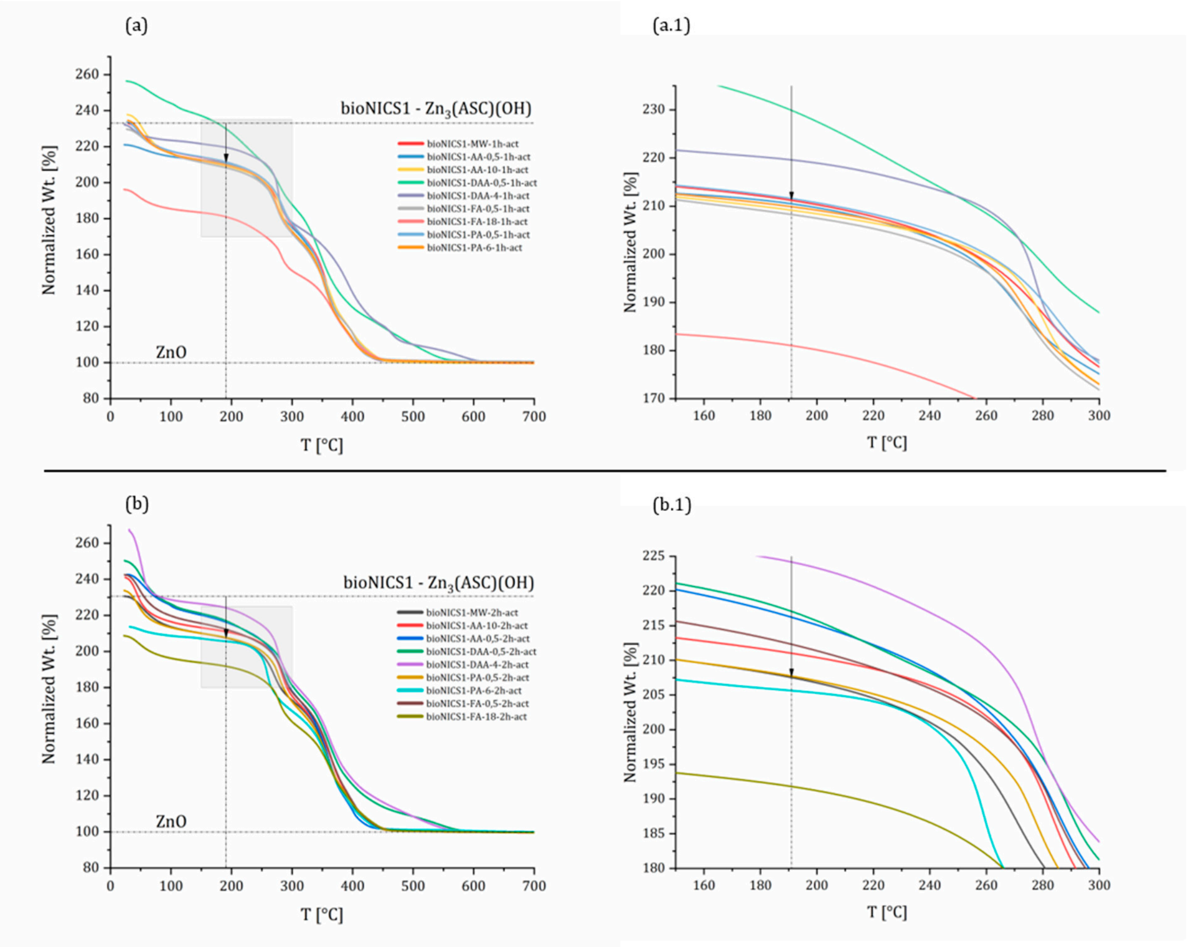

Figure S19: Comparison of thermogravimetric curves of selected, activated bioNICS1 materials crystalized under microwave heating for 1h (a) or 2h (b). Grey rectangle on (a) and (b) is magnified and presented in (a.1) and (b.1), where the line and arrow are set to the 191°C marking the start of degradation of the linker.

Table S1: Samples and their normalized inorganic residues.

Name of the sample	Normalized inorganic residue [%]
bioNICS1-FA-18-1h	53,81
bioNICS1-FA-0,5-1h	47,75
bioNICS1-PA-6-1h	47,32
bioNICS1-AA-0,5-1h	46,90
**bioNICS1-MW-1h**	46,89
bioNICS1-AA-10-1h	46,82
bioNICS1-PA-0,5-1h	46,72
bioNICS1-DAA-4-1h	45,30
bioNICS1-DAA-0,5-1h	41,48
bioNICS1-FA-18-2h	51,54
bioNICS1-PA-6-2h	48,55
**bioNICS1-MW-2h**	47,81
bioNICS1-PA-0,5-2h	47,71
bioNICS1-AA-10-2h	46,59
bioNICS1-FA-0,5-2h	45,96
bioNICS1-AA-0,5-2h	45,31
bioNICS1-DAA-0,5-2h	44,78
**bioNICS1-1day**	49,80
bioNICS1-6(fa)-1	49,37
bioNICS1-0,5(pa)-1	48,79
bioNICS1-0,5(fa)-1	48,42
bioNICS1-0,5(aa)-1	48,01
bioNICS1-6(pa)-1	47,90
bioNICS1-0,5(daa)-1	45,13
bioNICS1-16(aa)-1	42,68
bioNICS1-3(daa)-1	41,17
**bioNICS1-3days**	49,00
bioNICS1-6(fa)-3	48,53
bioNICS1-0,5(pa)-3	48,44
bioNICS1-0,5(fa)-3	47,87
bioNICS1-6(pa)-3	47,19
bioNICS1-0,5(aa)-3	44,97
bioNICS1-0,5(daa)-3	44,73
bioNICS1-16(aa)-3	41,80
bioNICS1-3(daa)-3	41,22

7. Study of defects

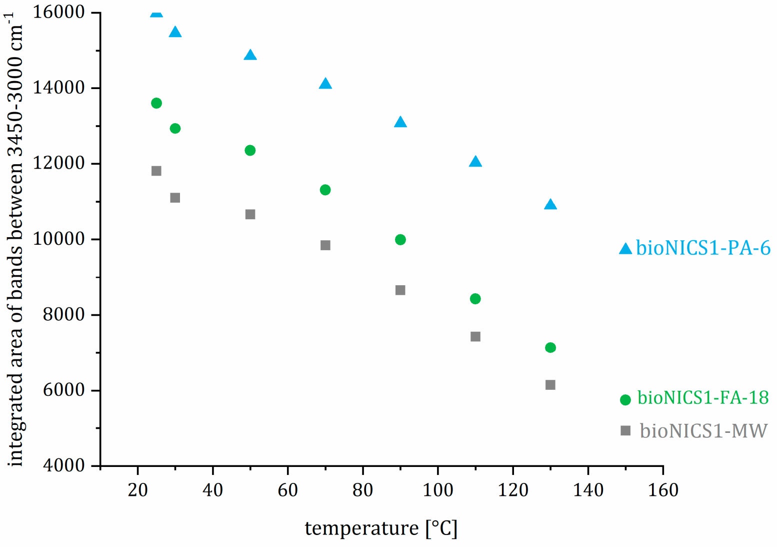

Figure S20: Integrated area of bands between 3450 – 3000 cm^{-1}.

DRIFTS clearly indicates the involvement of modulators within the structure. Their occurrence was therefore examined by liquid NMR of DCl digested both modified samples and pristine material for comparison.

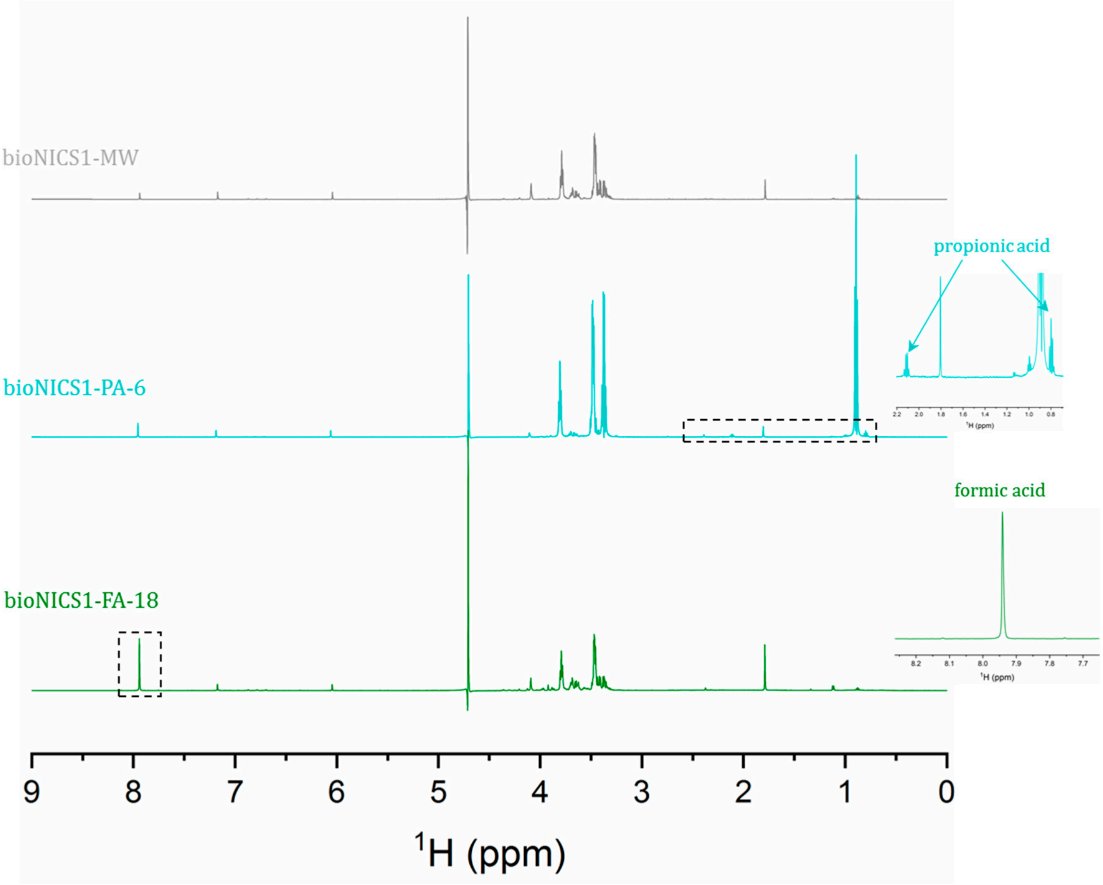

Figure S21: ¹H NMR spectra of the selected bioNICS-1 samples degraded in DCl solution. Insets show zoomed areas indicated by dashed squares with the shifts corresponding to propionic and formic acid respectively. Remaining signals are due to the presence of solvent moieties and ascorbic acid-derived products.

Lower magnitude of weight loss in the bioNICS1-FA-18 sample does suggest linker deficient structure, however, decrease of specific surface area points towards blocked or filled pores. Comparison of TG and DTG curves of synthesized and activated material shows, that there is a large amount of Zn-formate present in the pores of synthesized material, which is at least partially degraded to ZnO during the activation process. We see that in the increase of ZnO residue in the case of activated material. Initial presence of Zn-formate in synthesized material was confirmed by PXRD.

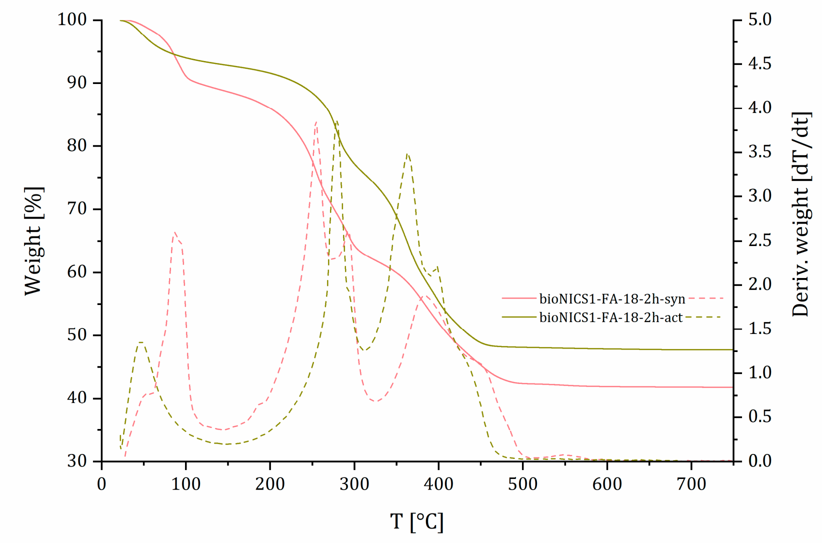

Figure S22: Comparison of thermogravimetric curves of bioNICS1-FA-18-2h material before (syn) and after (act) activation process.

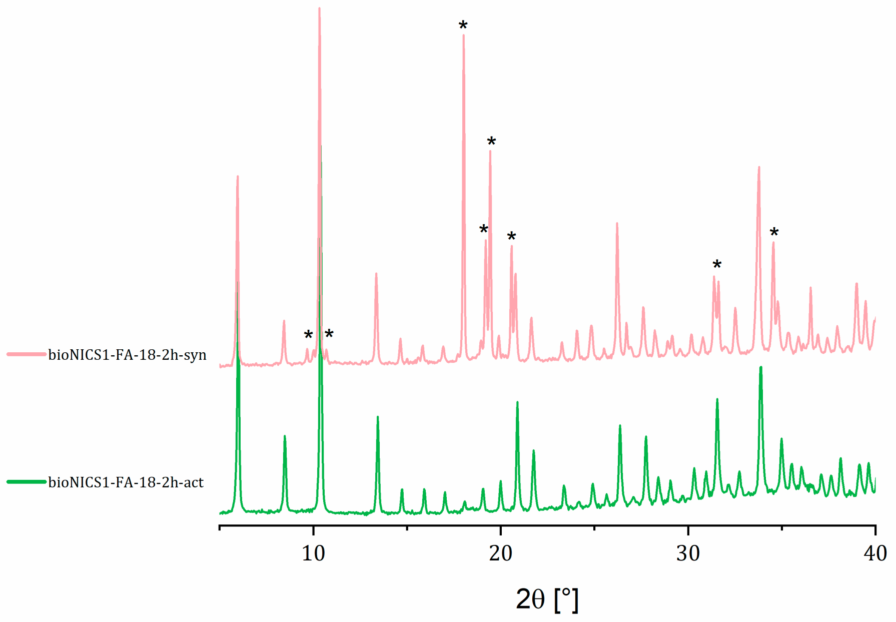

Figure S23: PXRD diffraction pattern of synthesized and activated bioNICS1-FA-18-2h. Indication most significant reflections corresponding to Zh-formate are marked with asterisk.

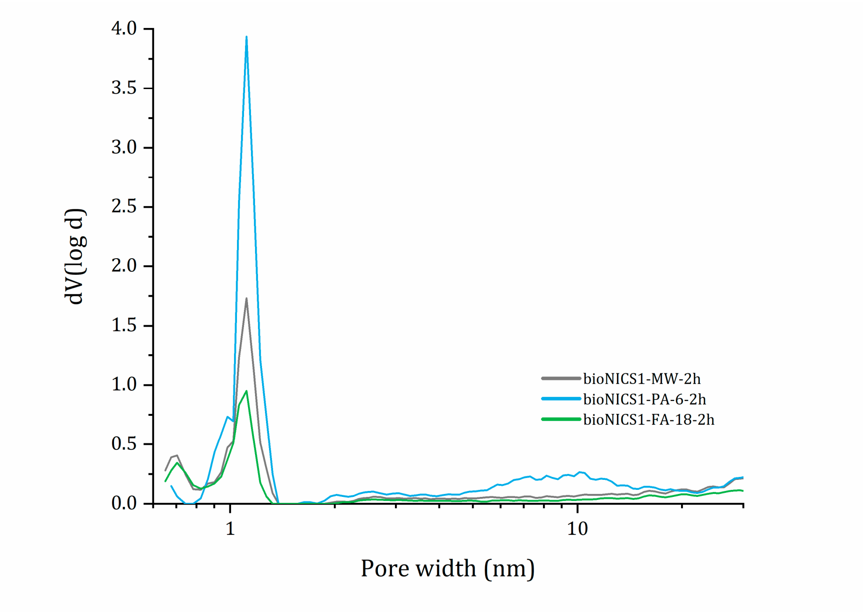

Figure S24: Comparison of pore size distribution of selected samples.

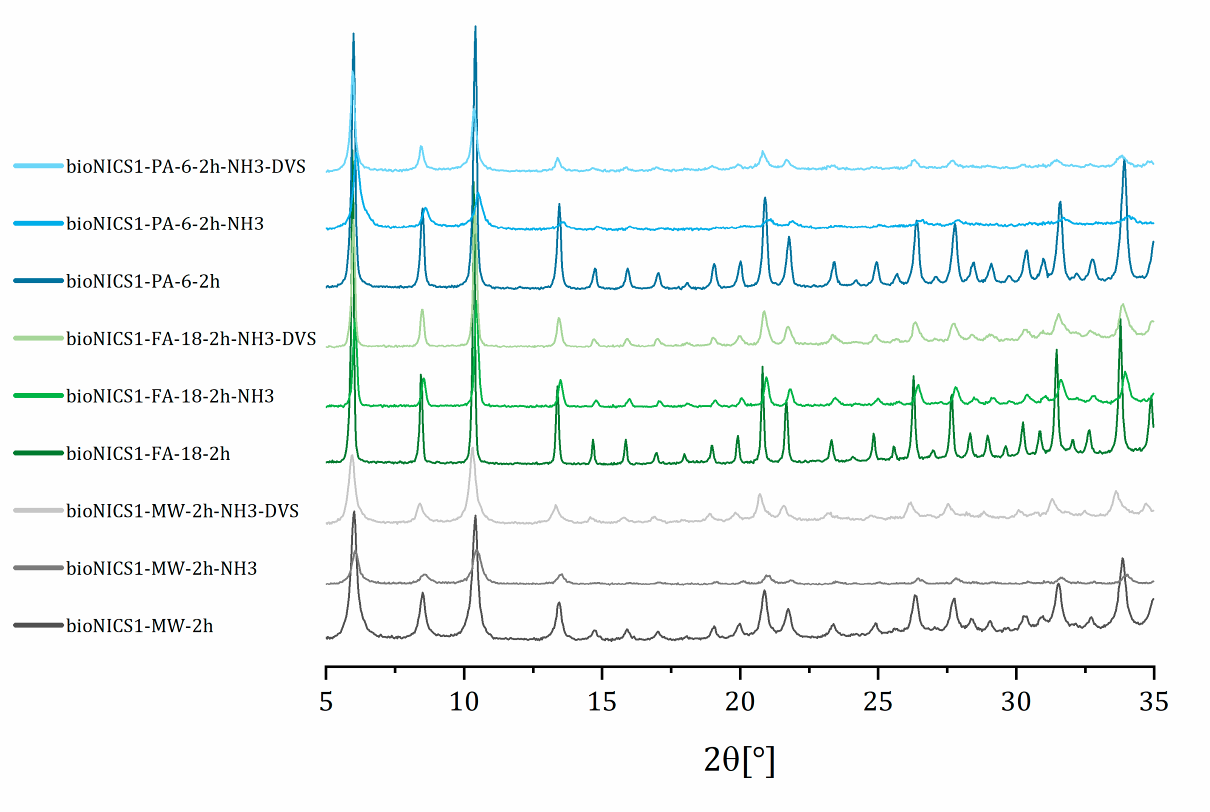

Figure S25: Comparison of PXRD difractograms confirming the original bioNICS1 structure of modulated and unmodulated before and after FTIR and DVS experiments.
